# Red Blood Cell-Encapsulated Nanoparticles for Long-Circulating, Improved Specificity Functional MRI

**DOI:** 10.1021/cbmi.5c00190

**Published:** 2026-01-12

**Authors:** Elizabeth J. Fear, Antonella Antonelli, Pasant Abdalla, Isaac Watson, Riccardo Di Corato, Victoria Annis, Oliver J. Mundell, Simon B. Duckett, Luigia Rossi, Elisa Zamboni, Mauro Magnani, Aneurin J. Kennerley

**Affiliations:** † Department of Biomolecular Sciences, University of Urbino Carlo Bo, Urbino 61029, Italy; ‡ Department of Neurosciences, Imaging and Clinical Sciences, University “G. D’Annunzio” of Chieti-Pescara, Chieti 66100, Italy; § Institute for Advanced Biomedical Technologies, University “G. D’Annunzio” of Chieti-Pescara, Chieti 66100, Italy; ∥ Biomedical Imaging Science Department, Leeds Institute of Cardiovascular and Metabolic Medicine, 4468 University of Leeds, Leeds LS2 9JT, U.K.; ⊥ Institute for Microelectronics and Microsystems (IMM), CNR, Via Monteroni, Lecce 73100, Italy; # Center for Biomolecular Nanotechnologies, Istituto Italiano di Tecnologia, Arnesano 73010, Italy; ¶ Department of Chemistry, 8748 University of York, York YO10 5DD, U.K.; ∇ Department of Sports & Exercise Science, 5289 Manchester Metropolitan University, Manchester M15 6BH, U.K.; ○ Wolfson ACTIVE Imaging Laboratory, Institute of Sport, Manchester Metropolitan University, Manchester M15 6BH, U.K.; ⧫ School of Psychology, 6123 University of Nottingham, Nottingham NG7 2RD, U.K.

**Keywords:** super paramagnetic iron oxide nanoparticles (SPIONs), red blood cells (RBCs), nanocarriers, encapsulation, functional imaging, preclinical, contrast

## Abstract

Nanoparticle-based MRI contrast agents have shown promise for advancing noninvasive imaging, but their clinical utility is limited by rapid clearance, poor biocompatibility, and lack of sustained signal. Here, we present a red blood cell (RBC)-based nanocarrier platform that encapsulates superparamagnetic iron oxide nanoparticles (SPIONs) following hypotonic dialysis and resealing of the cell membranes. This biomimetic “Trojan horse” strategy exploits the inherent circulation time, deformability, and biocompatibility of RBCs to prolong the nanoparticle lifetime and enhance the translational potential. In vivo rodent studies demonstrated that SPION-loaded human RBCs provide robust, long-lasting cerebral blood volume (CBV)-weighted functional (f) MRI signal with >5-fold magnitude stronger responses over conventional/established blood oxygenation level-dependent (BOLD) contrast. In addition, functional brain mapping using cell encapsulated SPIONs show improved laminar specificity, with activity localized to cortical layer IV. Compared with free SPIONs, loaded cells achieved >30 min of stable *T*_2_* contrast at one-quarter of the iron dose, while maintaining physiologically plausible CBV maps. These findings confirm efficacy and establish RBC encapsulation as a versatile and biocompatible nanomedicine platform for extending nanoparticle circulation and enabling high-resolution functional imaging with broad implications for translational applications in neurology, oncology, and theragnostics.

## Graphical abstract

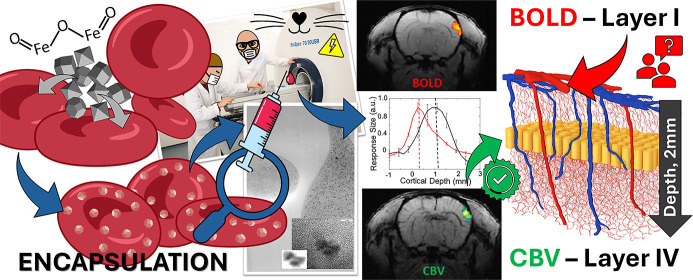

## Introduction

Nanoparticle-based contrast agents have been widely explored for enhancing functional biomedical imaging, including for brain mapping,^1,2^ yet their translation to the clinic has been limited by rapid clearance, toxicity concerns, and inconsistent pharmacokinetics.^3–6^ Superparamagnetic iron oxide nanoparticles (SPIONs), in particular, offer high magnetic susceptibility and well-characterized relaxivity, but free SPIONs are rapidly sequestered by the reticuloendothelial system, necessitating high doses that limit safety and reproducibility.^2,7–10^ A long-standing challenge in nanomedicine is therefore to develop delivery strategies that extend the circulation time, preserve nanoparticle function, and provide reliable contrast in vivo. This has motivated the development of biomimetic^11–14^ and polymeric^15–17^ delivery architectures to overcome clearance and pharmacokinetic barriers for SPION-based contrast in MR. These rational designs can be further functionalized providing powerful structural and pathological information via imaging to study disease progression.^18–20^ Among these approaches, leveraging living cells as carriers offers an especially powerful and biologically integrated strategy, providing capabilities that surpass those of synthetic biomimetic or polymeric platforms.^21^

Red blood cells (RBCs) offer an attractive, novel solution to the challenges outlined above. As the body’s natural oxygen carriers, RBCs circulate for up to 120 days, maintain exceptional biocompatibility, and possess unique deformability that enables microvascular access. Previous studies have demonstrated the feasibility of using RBCs as carriers for drugs and imaging agents, suggesting a generalizable strategy for improving nanoparticle pharmacokinetics and reducing immune clearance.^22^ By encapsulating SPIONs within RBCs, it becomes possible to combine the sensitivity of magnetic nanoprobes with the stability and safety of an autologous cell carrier, yielding a translationally relevant biomimetic platform.^6,23–28^ This “Trojan horse” strategy transforms the circulation lifetime of SPION-based contrast agents from minutes to potentially weeks, enabling sustained, biocompatible MR imaging with strong paramagnetic contrast.^29^

Here, we report the development and characterization of SPION (specifically Ferucarbotran)-loaded human RBCs (FLH-RBCs) as a long-circulating functional (f)­MRI contrast agent. We show that encapsulation preserves RBC morphology and function while providing sustained *T*_2_*contrast in an in vivo rat model at reduced iron doses compared to that of free nanoparticles. Rat RBCs are less deformable, more rigid, and prone to membrane damage and crystallization during osmotic treatments,^30–32^ we therefore employed bioengineered human RBCs for cross-species delivery. Our prior work has confirmed the stability and tolerability of this approach for both mouse^26^ and human^28^ RBCs through in vivo pharmacokinetics. These biomimetic Ferucarbotran-loaded RBCs also find application in development of Magnetic Particle Imaging-based diagnostics.^33^

As a proof of application, we importantly demonstrate that circulating FLH-RBCs enable high-resolution, cerebral blood volume (CBV)-weighted functional (f) MRI in rats, revealing laminar-specific neurovascular responses that exceed (>5 fold) conventional Blood Oxygenation Level Dependent (BOLD) amplitudes. Somatosensory responses peaked in cortical layer IV, the primary site of thalamocortical input, demonstrating enhanced laminar specificity compared to conventional/established blood oxygenation level-dependent (BOLD), which typically localizes to superficial cortical vasculature.^34,35^

These findings establish RBC encapsulation as a versatile nanocarrier technology for extending nanoparticle circulation and enabling advanced biomedical imaging. By transforming blood into a long-circulating, paramagnetically enhanced imaging agent, our platform offers a clinically viable path to high-resolution CBV-weighted fMRI.

For context, CBV changes are an extremely important parameter when considering brain physiology and pathophysiology.^36^ Abnormal CBV responses have been associated with central nervous system (CNS) disorders including stroke^37,38^ and Alzheimer’s disease.^39–41^ In addition, CBV mapping has also shown potential for investigating hemodynamic abnormalities that are associated with inflammatory activity, lesion reactivity, and vascular compromise in multiple sclerosis lesions.^42^ Broader applications in oncology see CBV measures used for quantitative assessment of angiogenesis^43^ and tumor vascularization.^44^ Therefore, accurate mapping of CBV changes allows differentiation to present between damaged/recoverable tissue as an important biomarker when considering development of CNS drugs or making other informed critical treatment decisions. As such, there exists a need for approaches which provide CBV weighted measures of neuronal function, that are easy to implement on all MRI scanners, and provide improved spatiotemporal mapping with higher CNR over traditional BOLD fMRI methods.

Intravascular contrast agents, utilizing superparamagnetic iron oxide nanoparticles (SPIONs) have been used in preclinical models for CBV-weighted fMRI.^1,2^ These agents are generally maghemite (γ-Fe_2_O_3_)- or magnetite (Fe_3_O_4_)-based nanoparticles with hydrodynamic diameters ranging between 50 and 180 nm (core diameters <10 nm).^7^ They are often coated with biocompatible polymers such as dextran, carboxydextran, and polyethylene glycol.^8^ With the same coating material, smaller particles <50 nm hydrodynamic diameter often referred to as ultrasmall (U)­SPIONs have a longer blood half-life.^3^ In terms of functional brain mapping, at sufficiently high doses, the magnetic susceptibility of SPIONs overrides the comparatively smaller susceptibility effects caused by blood oxygen saturation changes during neuronal activity. Resulting signal changes are therefore a direct reflection of CBV changes, as blood vessels dilate, increasing plasma volume and, therefore, more paramagnetic agent into the voxel of interest.

Preclinical fMRI data demonstrates that the high CNR signal proffered through use of intravenous SPION-based contrast agents delivers functional sensitivity at cortical laminar resolutions.^2,9,10^ The use of i.v. injection of SPIONs has the practical advantage that blood magnetization is optimized at any echo time and any magnetic field; therefore, short echo times may be used and, overall, this enables high CNR.^2^ Unfortunately, application in clinical settings remains limited since SPIONs have the disadvantage of becoming rapidly tagged by serum proteins^4^ for elimination by phagocytes as part of the body’s immune defense system, the reticuloendothelial system (RES). This short half-life (on the order of minutes)^3^ combined with the risks of iron accumulation and potential cytotoxicity^6^ are the primary limitations of clinical translation. The FLH-RBCs solution detailed here overcomes these limitations.

## Results and Discussion

This interdisciplinary study bridging biochemistry and imaging physics demonstrates the transformative clinical potential of bioengineered contrast agents—specifically, superparamagnetic iron oxide nanoparticles (SPIONs) encapsulated within human red blood cells (RBCs) using hypotonic dialysis followed by membrane resealing (Figure 1). This procedure yielded stable SPION-loaded RBCs (FLH-RBCs) with high encapsulation efficiency while maintaining cell recovery rates comparable to those of untreated controls. By combining endogenous biocompatibility with enhanced magnetic susceptibility, this “Trojan horse” strategy overcomes key limitations of conventional BOLD fMRI and nanoparticle-based diagnostics, offering a clinically viable pathway for sensitive detection of neurovascular dysfunction.

**Figure 1:**
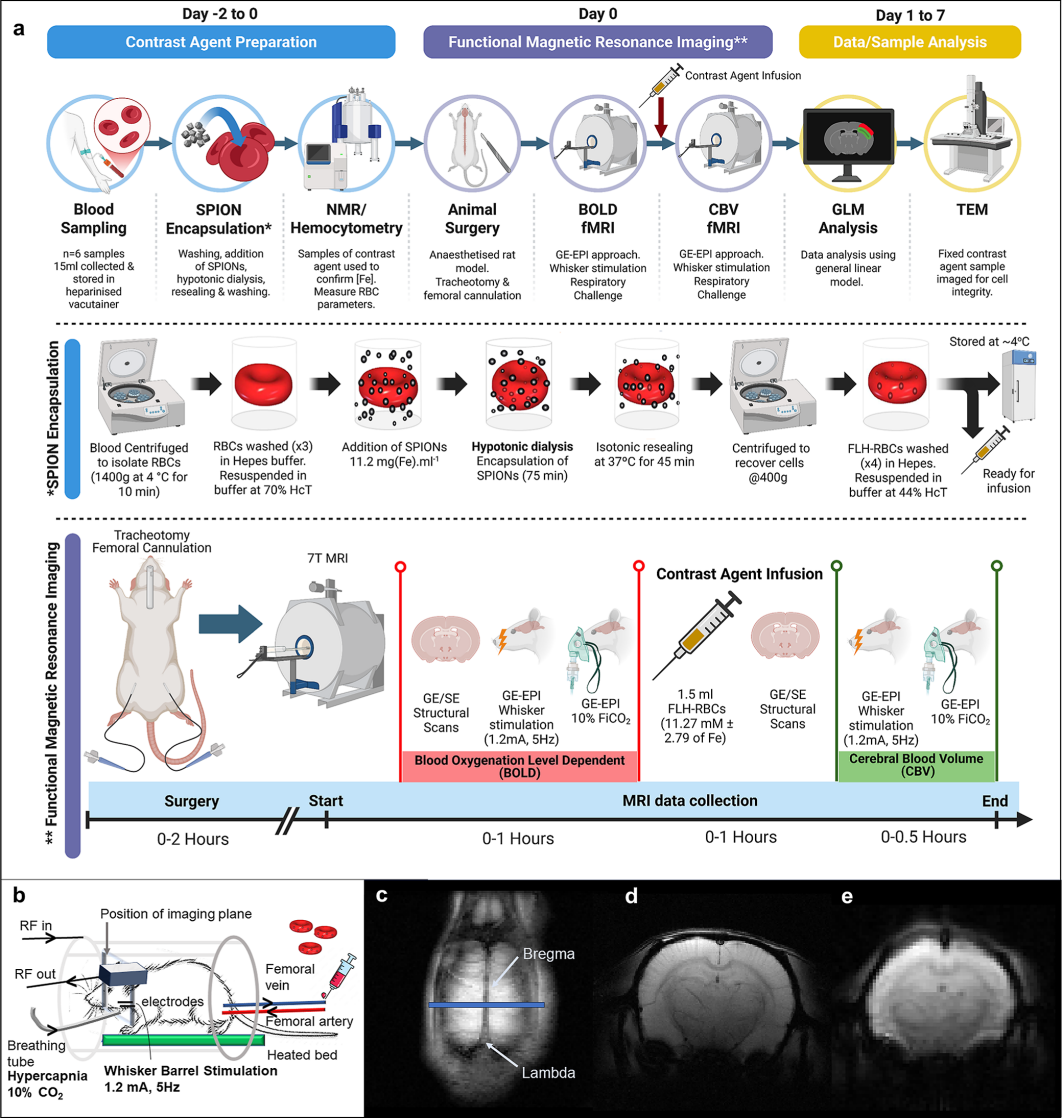
Experimental scheme depicting the general fabrication of the RBC-encapsulated SPION contrast agent for functional (f) MRI experiments. The pipeline covers the three main components of this broad study, contrast agent preparation, functional magnetic resonance imaging, and the post experiment data/sample analysis (a). A schematic representation of the SPIONs loading into RBCs by hypotonic dialysis and isotonic resealing is provided along with specific timings of the in vivo part of this work. Preclinical fMRI set up (b) showing the positioning of the imaging plane, RF volume transmit coil, RF head receiver coil, femoral artery and veins cannulated to monitor blood pressure and infuse the CA, breathing tube for ventilation and delivery of medical air/CO_2_ for hypercapnia respiratory challenge and the positioning of the electrodes for electrical whisker stimulation. (c) GE in the coronal plane of the dorsal surface of the brain showing bregma and lambda and the positioning of the imaging plane 3 mm posterior to bregma (TR/TE = 300/4 ms, 128*128, FOV = 30 mm^2^, slice thickness = 1 mm, FA = 90°, slices = 1). (d) GE in the axial plane (TR/TE = 1000/10 ms, 256 × 256, FOV = 30 mm^2^, slice thickness = 1 mm, FA = 90°, slices = 9). (e) GE -EPI in the axial plane (TR/TE = 1000/12 ms, 96 × 96, FOV. This scheme was developed using BioRender.

Full loading protocols are provided in the Supporting Information methods. Briefly, RBCs are isolated from drawn human blood by centrifugation, and the serum and buffy coat are removed. The packed cells are washed in Hepes buffer and then resuspended in the same buffer at 70% hematocrit (HcT). For SPION encapsulation, cells are subjected to hypotonic dialysis in the presence of Ferucarbotran (11.2 mg (Fe)/1000 μL RBCs at 70% Hct) at 4 °C for 75 min. Ferucarbotran is composed of maghemite, ferric oxide (γ-Fe_2_O_3_) and magnetite, ferroferric oxide (Fe_3_O_4_) with a carboxydextran coating and a mean hydrodynamic diameter of 57 nm; [56 mg Fe/ml or 1 M Fe]. After the dialysis process, the RBCs undergo isotonic resealing at 37 °C for 45 min thus obtaining Ferucarbotran-loaded human RBCs (FLH-RBCs). The resealed cells are recovered by centrifugation and washed four times with Hepes buffer to remove the nonencapsulated magnetic nanomaterial. The FLH-RBCs are then resuspended in Hepes buffer at 44% HcT ready for direct in vivo infusion, or alternatively can be stored at 3–4 °C for up to 2–3 days.^29^ Unloaded (UL-RBCs) controls are prepared following the same dialysis procedure but in the absence of the SPIONs.

It is noted that not all SPIONs are equally suitable for encapsulation in human RBCs. The suitability is dependent on the size, synthesis method, and coating of the particles. If a SPION is uncoated or not stabilized, it may absorb onto the RBC membrane through electrostatic attraction or hydrophobic interactions. Nanoparticles depending on their size, coating, and charge can change RBC morphology, e.g., echinocyte formation (RBCs take on a spiky appearance), membrane stiffening, and alteration of shape. Appropriate coating prevents iron leakage from the core and prevents hemoglobin oxidation or the generation of reactive oxygen species. Uncoated or highly charged SPIONs may also cause hemolysis at a high concentration or leakage of hemoglobin. The size of the nanoparticles is also important, as they must be small enough to pass through the enlarged membrane pores during the hypotonic dialysis phase. In addition, the use of high concentrations of particles during the loading procedure can result in the formation of clusters and eventual binding to the RBC membrane. The material used (Ferucarbontran) and the procedures reported in this paper were selected by comparing the performance of several nanoparticles.^45^ Ferucarbotran has a carboxydextran coating, which stabilizes the nanoparticles by preventing them from aggregating. The carboxyl groups within the coating give the nanoparticles a net negative surface charge, which prevents immediate strong electrostatic attraction to the negatively charged RBC membrane and unwanted binding.

### Preservation of Red Blood Cell Morphology and Function

To confirm that SPION encapsulation did not compromise RBC physiology, we evaluated the cellular morphology, deformability, and osmotic fragility. Microscopy revealed intact biconcave shapes, while the hemoglobin content and membrane integrity were preserved. Specifically, optical imaging confirmed that native cell integrity is maintained following the RBC loading procedure (Figure 2a,d). Transmission electron microscopy (TEM) images demonstrated a clear homogeneous distribution of the iron oxide nanoparticles within the cell cytoplasm of the loaded RBCs (Figure 2e,f). Importantly, there is no adhesion of nanoparticles to the cell membrane which could trigger an immune response and the nanoparticles are distributed within the RBCs in a homogeneous manner. High resolution TEM observations (Figure 2f1) showed the lattice planes inside the nanoparticles with spacings (*d* = 0.295 nm and *d* = 0.298 nm) consistent with magnetite, ferroferric oxide Fe_3_O_4_ (d(220) = 0.2967 nm) and maghemite, ferric oxide Fe_2_O_3_ (*d*(220) = 0.2953 nm) crystal structures, confirming nanoparticle identity.

**Figure 2:**
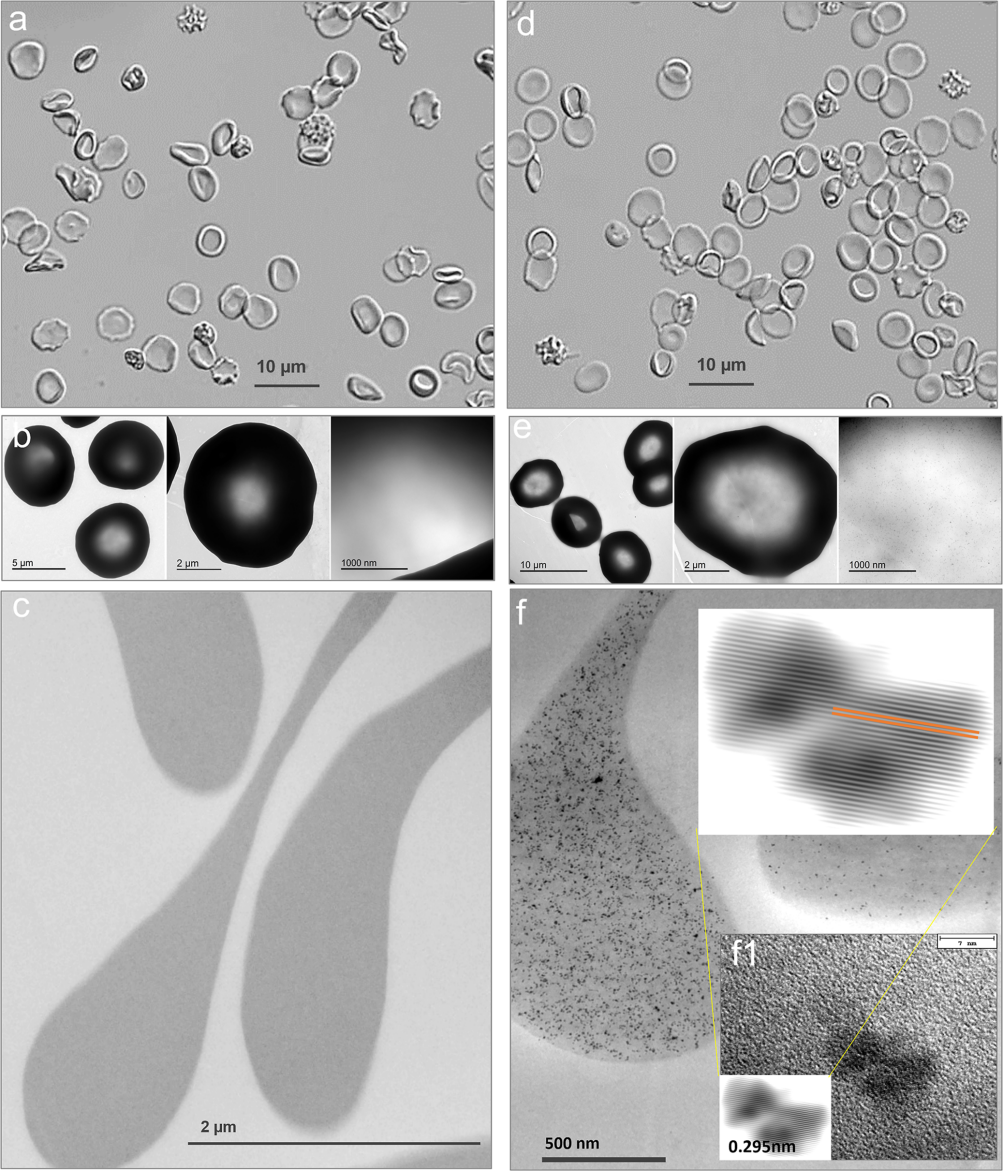
Microscopy images of loaded and unloaded red blood cells. Human unloaded RBCs (a–c): (a) optical microscope image, b and c) TEM analyses of whole and slice cells, respectively. FLH-RBCs (d–f): (d) optical microscope image, e and f) TEM analyses of whole and slice cells, respectively. The insert (f1) obtained by high resolution electron microscope shows the crystalline state of the dispersed nanoparticles after denoising using Fourier transform (FFT). The lattice planes of the nanoparticle can be clearly distinguished and are highlighted in orange for convenience.

Evaluation of core cell integrity metrics of Ferucarbotran loaded human (FLH)-RBCs were completed relative to native, nondialyzed (ND), and unloaded (UL) RBCs. A typical cell recovery of loaded RBCs ranged from 65% to 72%, similar to that of UL-RBCs, consistent with previously reported studies^23,24^ (see Table 1). Although loading (FLH-RBCs) resulted in smaller cell volume (MCV 66 ± 5 vs 86 ± 5 fL for ND-RBCs) and reduced mean hemoglobin content (MCH 18 ± 1 pg), the mean cellular hemoglobin concentration (MCHC 27 ± 1 g/dl) remained within a near-physiological range (MCHC 33 ± 4 g/dl for ND-RBCs). These values are in line with prior reports involving mammalian RBC encapsulation procedures,^46,47^ including human cells.^48,49^

**Table 1:** NMR Measurements of *T*_1_ and *T*_2_ of FLH-RBCs and Their Biological Parameters

samples	*T*_1_ (ms)	*T*_2_ (ms)	Fe [mM]	MCV (fl)	MCH (pg)	MCHC (g/dl)	cell recovery (%)
ND-RBCs *n* = 6	2173 ± 214	64 ± 3	/	86 ± 5	30 ± 4	36 ± 5	/
UL-RBCs *n* = 3	2022 ± 149	60 ± 8	/	72 ± 5	24 ± 2	33 ± 4	71 ± 7
FLH-RBCs *n* = 3	74.4 ± 31.1	<5	11.3 ± 4.8	66 ± 5	18 ± 1	27 ± 1	73 ± 8

These results demonstrate that SPION encapsulation maintains the structural and functional properties of the RBCs.

### Magnetic Properties and Relaxivity of Encapsulated SPIONs

Next, we characterized the magnetic performance of the encapsulated nanoparticles. FLH-RBCs exhibited robust *T*_2_ and *T*_2_* relaxivity, with values comparable to or exceeding those of free SPIONs (see Table 1). FLH-RBCs showed a marked reduction in *T*_1_ values (74.4 ± 31.1 ms) and undetectably short *T*_2_ values (<5 ms) compared to controls (*T*_1_: 2022 ± 149 ms for UL-RBCs; 2173 ± 214 for ND-RBCs). The mean final concentration of magnetic material in FLH-RBCs samples was estimated at 11.27 mM ± 4.83 (std dev) and 2.79 (std err), corroborated by ICP-OES (10.8 ± 3.8 mM). This represents an approximate 5–7% efficiency of SPION loading into the RBCs. Importantly, the relaxivity remained stable across repeated measurements, confirming that encapsulation did not diminish the magnetic properties required for MRI applications.

### In Vivo Pharmacokinetics and Contrast Persistence

We then investigated circulation dynamics and contrast longevity in vivo. FLH-RBC suspensions were injected (IV) in a rodent model and tested as a suitable contrast agent for CBV-weighted fMRI. We compared in vivo retention between FLH-RBCs and free Ferucarbotran SPIONs. Following infusion, both agents produced an initial ∼20% drop in the *T*_2_* weighted gradient echo (GE)-EPI signal. However, signal loss recovered rapidly after free SPIONs (half-life 12.5 s), consistent with RES clearance^26^ (Figure 3a–c). In contrast, FLH-RBCs achieved a sustained *T*_2_ * signal drop (−15%) persisting for >30 min (Figure 3d–f), despite using only one-quarter of the iron dose, reflecting the protective and long-circulating properties of the RBC carrier. This suggests significantly improved pharmacokinetics via RBC encapsulation^23^ over conventional nanoparticle formulations. Our approach delivers dramatically reduced loss rate of SPIONs in circulation and unlocks longitudinal assessment critical for therapeutic and diagnostic purposes, further highlighting the improved efficacy of our “Trojan horse” strategy.

**Figure 3:**
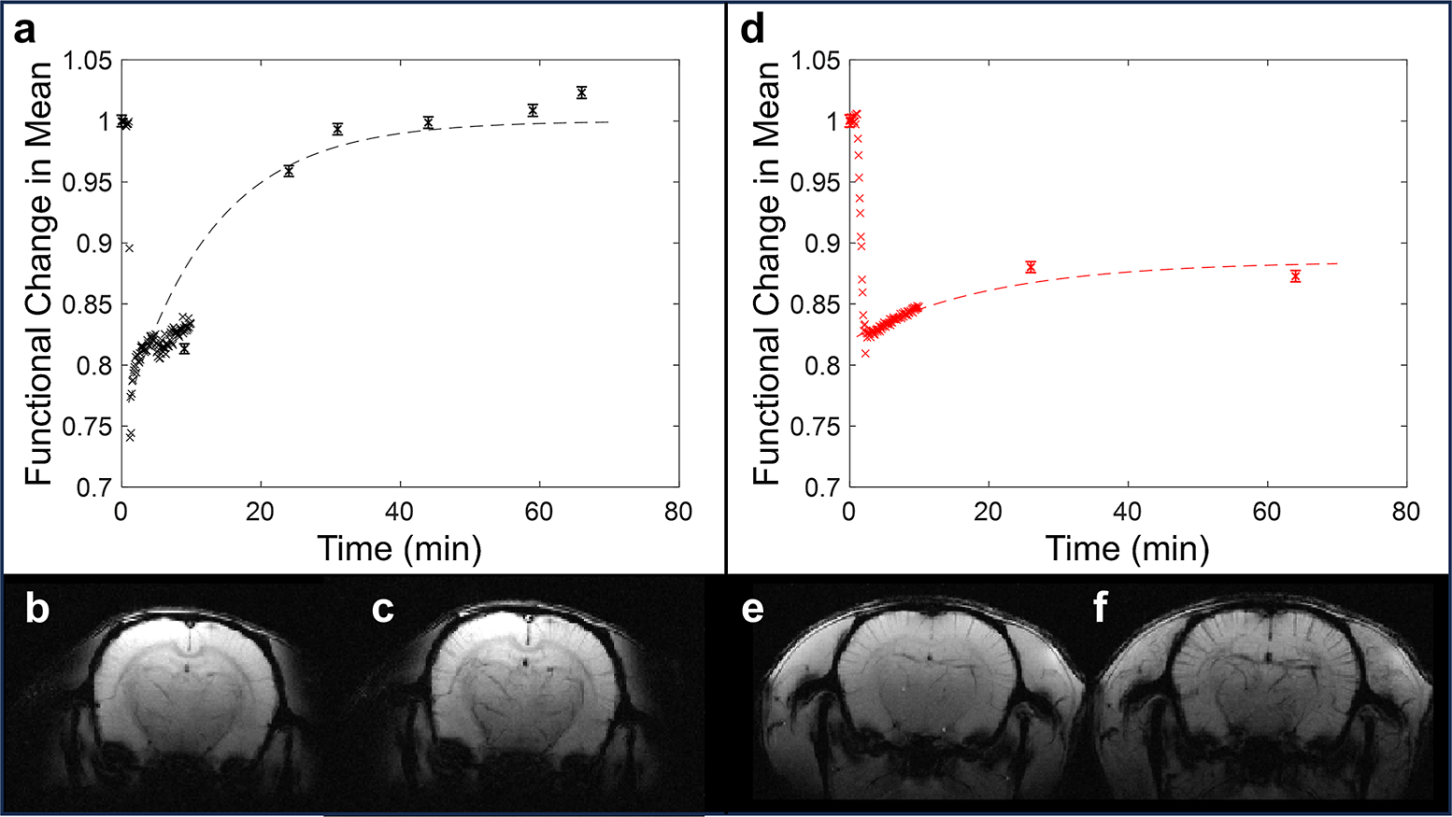
Blood clearance rate data for free and encapsulated SPIONS. Infusion and clearance of free ferucarbotran compared with FLH-RBCs measured during a GE-EPI (0–10 min) and high-resolution GE structural scan at 10–60 min (256 × 256 pixels, FOV = 30 mm, slice thickness = 1 mm, slices = 9, TR/TE = 1000/12 ms, flip angle = 90°, 2 averages)). (a) Infusion of free ferucarbotran (40 μmoles Fe) and clearance; (b) GE scan prior to injection of free ferucarbotran *t* = 0 min; (c) GE scan at *t* = 60 min after injection of free ferucarbotran; (d) infusion of FLH-RBCs (10 μmoles Fe) and clearance; (e) GE scan prior to injection of FLH-RBCs *t* = 0 min; (f) GE scan at *t* = 60 min after injection of FLH-RBCs.

In a subselection of animals, we quantified baseline blood volume fraction by analyzing R_2_* changes during stepwise FLH-RBC infusion (Supporting Information Figure S6). Applying a standard magnetic susceptibility constant (Δχ = 0.571 ppm based on the literature^44^) yielded implausibly low blood volume fraction estimates (*V*_f_ of ∼1%; Supporting Information -Figure S7a–c). Adjusting χ to 0.15 ppm—closer to the known susceptibility of hemoglobin (χ of Hbr is 0.157 ppm;^50^ Supporting Information Figure S8)— produced physiologically realistic values (∼6% in superficial cortex; ∼4% deeper), as shown in Figure 4. This adjustment highlights the importance of tuning susceptibility assumptions to encapsulated systems: increasing iron loading can help overcome oxygenation-induced changes in susceptibility that could confound the data. Note that free SPIONs could not be used for this analysis due to their rapid clearance.

**Figure 4:**
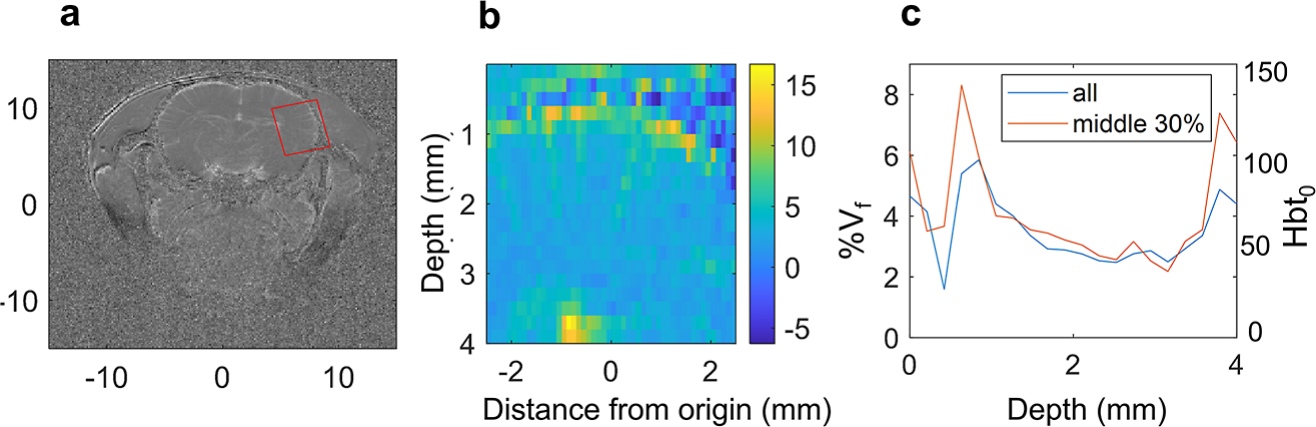
Blood volume fraction and concentration maps with FLH-RBCs. Blood volume fraction (*V*_f_) and concentration (Hbt_0_) maps with FLH-RBCs (16.4 mM) using magnetic susceptibility value Δχ = 0.15 (a–c). (a) Mean *V*_f_ map per voxel. (b) *V*_f_ calculated across a 2D cross section of whisker barrel cortex region. (c) Mean profile of the 2D cross section showing the mean *V*_f_ and Hbt_0_ per voxel across the cortex region (blue line) plus the central 30% of the selected area (red line).

### Functional MRI Demonstrates Layer-Specific Neurovascular Mapping

As a proof-of-application, we tested FLH-RBCs in functional (f)­MRI experiments. In rats, CBV-weighted fMRI revealed robust responses to whisker stimulation (9.4 ± 1.3%) and hypercapnia (37.1 ± 12.8%), exceeding BOLD amplitudes by more than 5-fold. Activation localized predominantly to cortical layer IV, confirming enhanced laminar specificity compared with superficial BOLD signals. Baseline CBV mapping further produced physiologically plausible blood volume fractions across the cortical layers.

Figure 5 shows representative coronal functional activity maps: BOLD, unprocessed signal following FLH-RBC injection, and CBV weighted, respectively, in response to whisker pad stimulation (5 Hz, 1.2 mA). A robust activation (2.5% BOLD change) was observed in the somatosensory barrel cortex. After FLH-RBC injection, we observed a negative contrast (∼−2.5%, as expected for a *T*_2_ * agent), and calculated CBV changes up to 12%.

**Figure 5:**
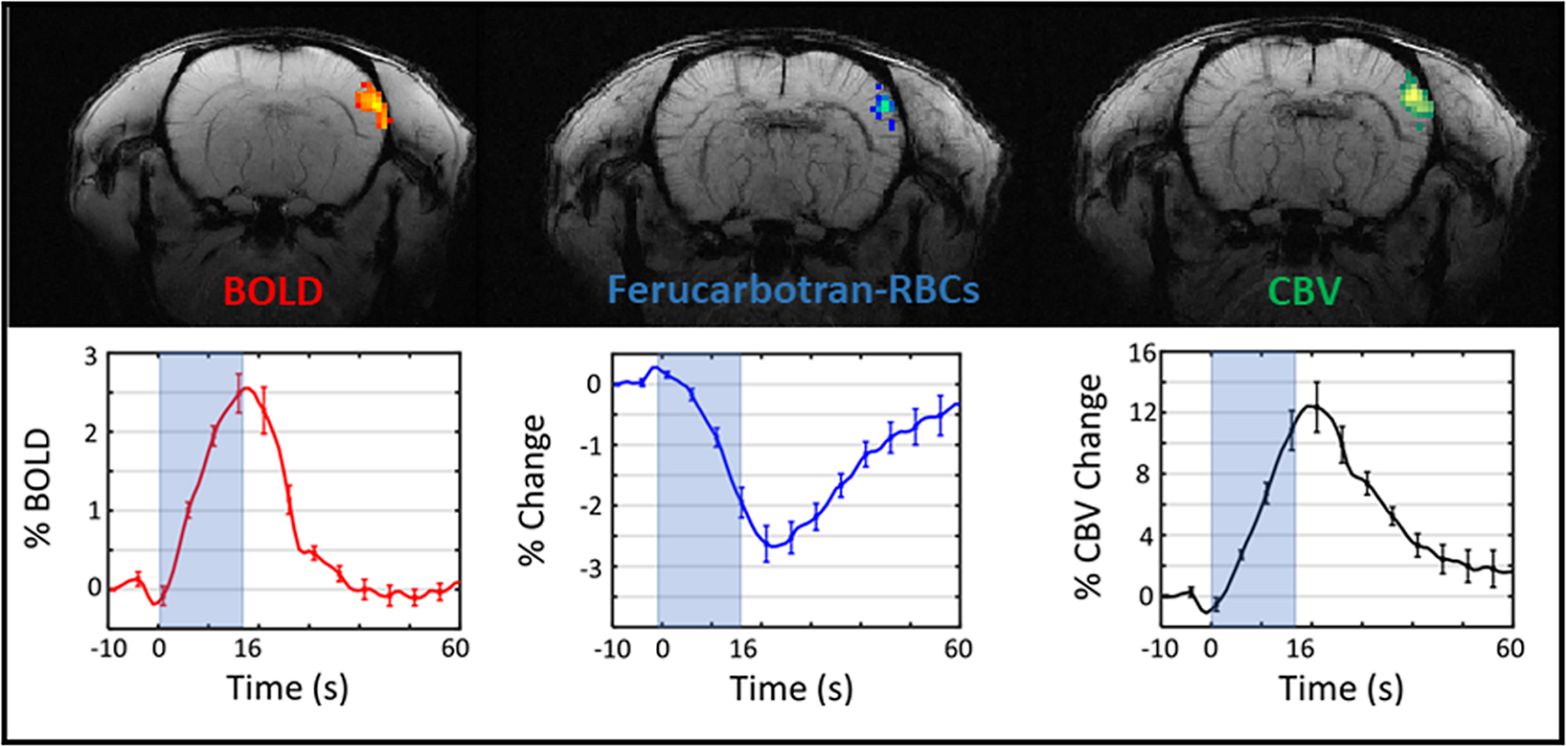
Representative pre and post functional MRI data (∼16.4 mM of FLH-RBCs). BOLD response (red) and the response after the injection of FLH-RBCs (blue) to somatosensory whisker stimulation (16 s, 1.2 mA, 5 Hz) and calculated CBV change (green), all in the whisker barrel cortex overlaid on GE structural scan. The corresponding time series for each condition are found beneath.

Figure 6 extends these results across five slices spanning the whisker barrel cortex, showing consistent CBV changes of 6–12%. These findings confirm that FLH-RBCs effectively enable CBV-sensitive imaging with a regional specificity. All multi slice data of BOLD, iron, and calculated CBV change for electrical whisker stimulus and respiratory challenges (whole brain) are shown in the Supporting Information (Supporting Information Figures S1–S5).

**Figure 6:**
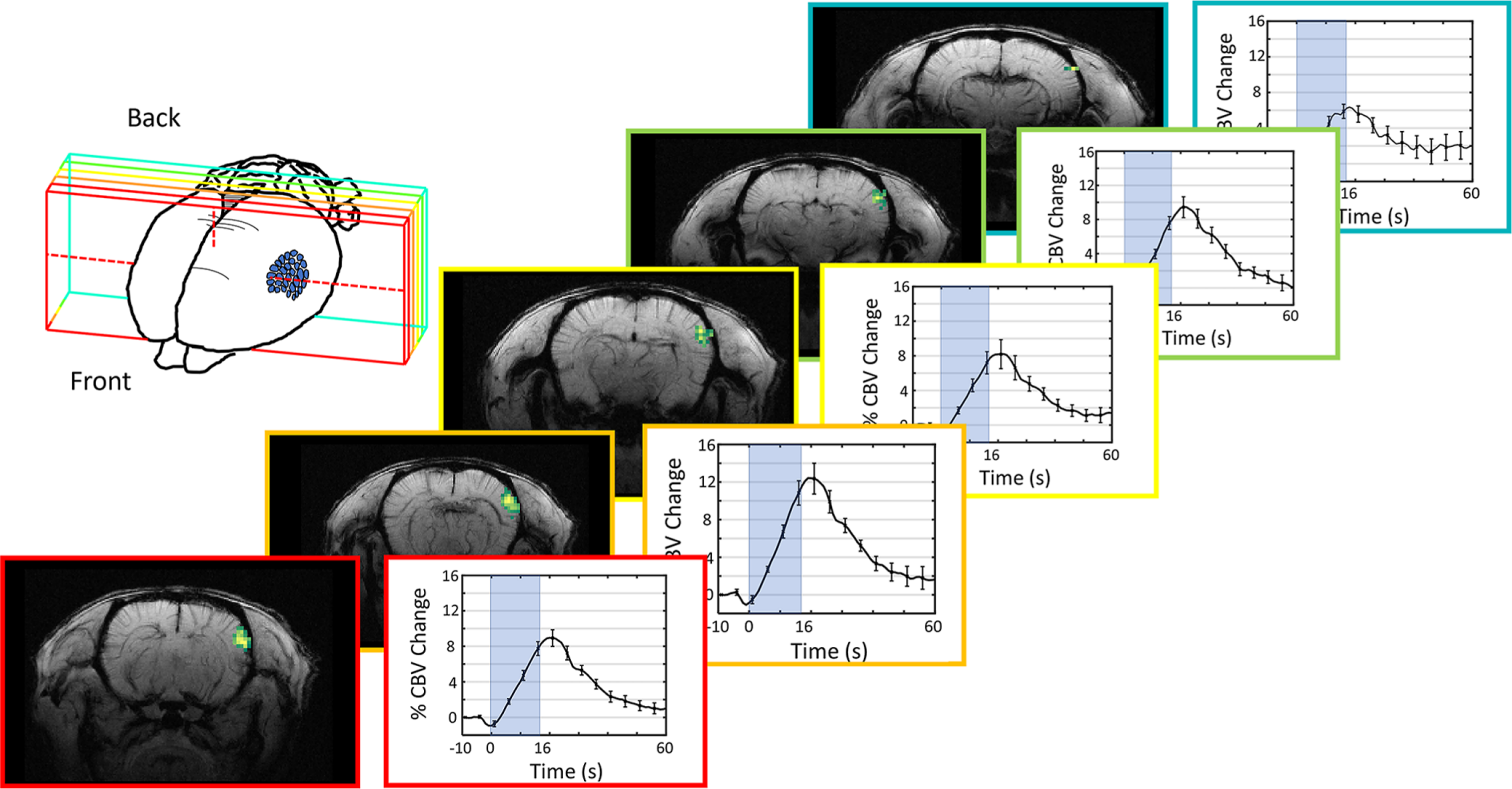
Representative CBV MRI data (∼16.4 mM of FLH-RBCs). CBV change in the whisker barrel cortex overlaid on GE structural scan in response to somatosensory whisker stimulation (16 s, 1.2 mA, 5 Hz) with corresponding percentage CBV time series for each of the 5 slices measured.

Group-level analyses showed peak BOLD (*n* = 7, all animals preinjection) and CBV (*n* = 3, after FLH-RBCs infusion) responses of 1.7 ± 0.3%, and 9.7 ± 1.1%, respectively, during whisker stimulation (Figure 7a). Notably, UL-RBCs controls (*n* = 3) produced no significant alteration in BOLD signals postinfusion (Figure 7a), indicating no perturbation of normal neurovascular functioning following human RBCs infusion. The inset in Figure 7a shows normalized signals. The preinjection and UL-RBC BOLD signal peaks are identical in shape, whereas the CBV peak shows evidence of delayed compliance, as expected.^10^

**Figure 7:**
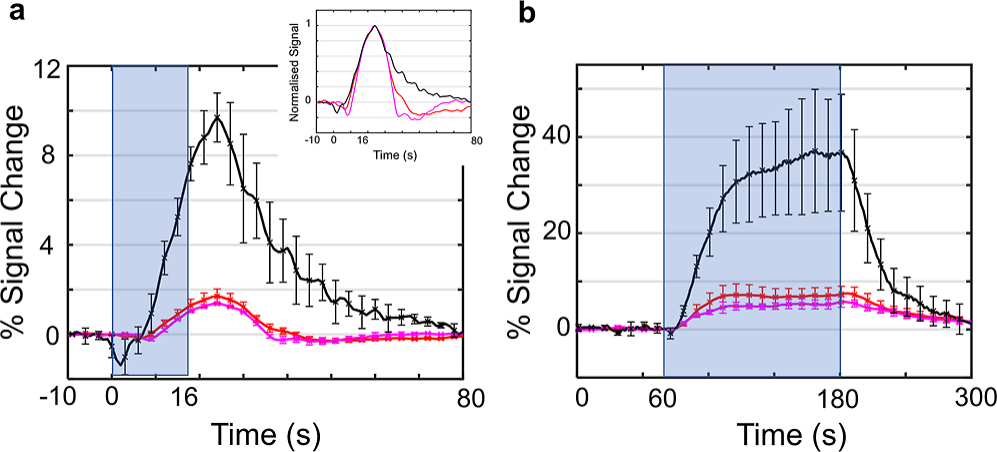
Mean BOLD response to somatosensory whisker stimulation in the whisker barrel cortex. Stimuli data showing a mean BOLD timeseries in the whisker barrel cortex in response to somatosensory whisker stimulation (16 s, 1.2 mA, 5 Hz, stimuli = 7) before infusion *n* = 7 rats (red), after infusion of only unloaded-RBCs (*n* = 3 rats, magenta) and mean calculated change in CBV time series after infusion of FLH-RBCS (black *n* = 3 rats). Insert-normalized signals; (b) mean BOLD timeseries in the cortex in response to hypercapnia (increased end-tidal FiCO_2_ < 10%) before infusion *n* = 7 rats (red), after infusion of unloaded-RBCs (*n* = 3, magenta), and mean calculated change in CBV time series after infusion of FLH-RBCS (black *n* = 3 rats).

Under hypercapnia challenges, CBV responses were also amplified relative to BOLD. CBV peaked at 37.1 ± 12.8%, compared to BOLD values of 6.8 ± 1.7% preinjection (*n* = 7) and 5.2 ± 1.0% post-UL-RBCs injection (*n* = 3) (Figure 7b). These findings confirm the sensitivity of FLH-RBCs to blood volume dynamics under both sensory and vascular stimuli and are consistent with previously reported changes in preclinical studies using free SPIONs in circulation.^35,51–55^

To evaluate the cortical resolution, we extracted cortical depth profiles from fMRI statistical maps. As shown in Figure 8, BOLD signal was maximal in superficial layers (0–0.5 mm), whereas CBV peaked at ∼1 mm depth, corresponding to layer IV—where thalamocortical inputs terminate. This laminar-specific activation is consistent with prior free SPIONs studies^35,56^ and spin–echo BOLD techniques,^57^ confirming the functional precision enabled by FLH-RBC contrast.

**Figure 8:**
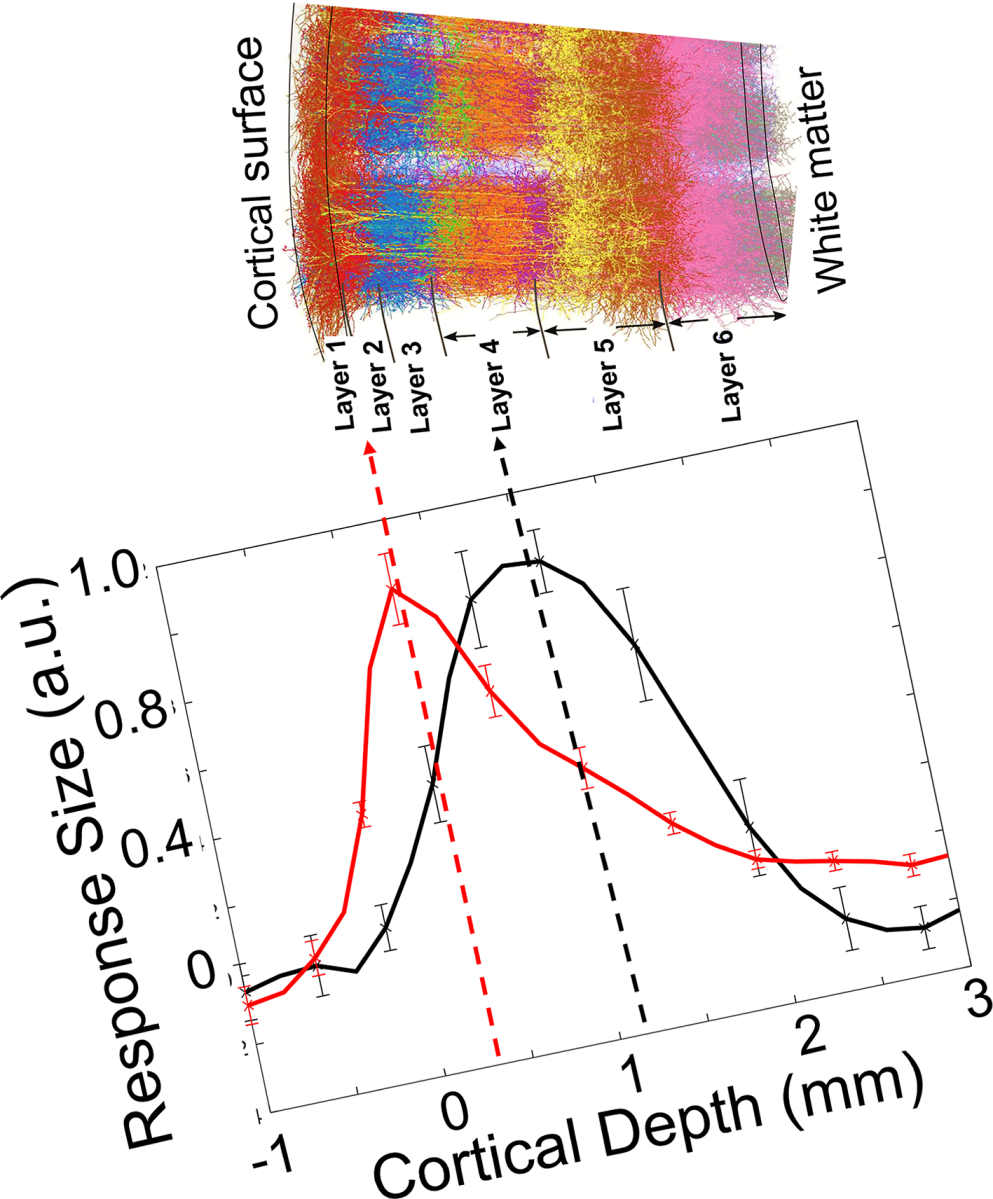
Mean BOLD and calculated CBV response across cortical depth. BOLD response (red *n* = 7) and the calculated CBV (black, using FLH-RBCs) from whisker stimulation across 3 animals underneath a representative 2D reconstruction of the six layers of the cortex in the rat brain.

### Implications for Functional Brain Mapping

Current clinical fMRI relies predominantly on the BOLD signal, which has limited spatial specificity due to superficial venous weighting and a low contrast-to-noise ratio (CNR). BOLD relies on changes in oxy/deoxy hemoglobin concentration, a secondary marker of neural activity that is heavily weighted toward large superficial cortical vasculature.^58–60^ This venous bias reduces spatial specificity and limits sensitivity to deeper brain structures and cortical lamina, where many disease processes originate. These constraints reduce its sensitivity to subtle but clinically important biomarkers of neuronal function/dysfunction, limiting diagnostic precision in conditions such as Alzheimer’s disease,^61,62^ vascular dementia, and stroke. Our bioengineered approach directly addresses these significant limitations: our FLH-RBCs method achieves 5–6 times greater signal amplitude than BOLD, offering a practical and scalable alternative with superior spatial fidelity.

We do note that alterative, non-BOLD fMRI techniques (e.g., GRASE,^63^ arterial spin labeling, ASL,^64^ steady state free procession SSFP,^65,66^ functional diffusion,^67^ phase-sensitive fMRI,^68,69^ inversion vascular space occupancy, VASO^70,71^ ) can also offer improved localization and physiological specificity.^58,59^ However, these methods are technically complex, yield lower contrast-to-noise ratio (CNR),^34,72^ and remain difficult to implement in clinical workflows. As a result, gradient-echo echo planar imaging (GE-EPI) BOLD remains the clinical standard despite its shortcomings.

The FLH-RBCs method maintains full compatibility with standard GE-EPI sequences routinely used in clinical practice, therefore bypassing the need for alternative, specialized CBV measures (e.g., vascular space occupancy) that require complicated bespoke MR pulse sequences.^34,71^ Moreover, it delivers CBV-weighted signal changes of 9.4 ± 1.3% during whisker stimulation and 37.1 ± 12.8% under hypercapnia, values that match or exceed those reported by established preclinical benchmarks using AMI-227,^35,51–55^ while offering a safer delivery mechanism.

The superior spatial specificity achieved with our contrast agent represents a major clinical advancement. We observed maximal CBV changes in cortical layer IV (∼1 mm from cortical surface), the canonical thalamocortical input zone, contrasting with the superficial (layer I, 0–0.5 mm) peak observed in BOLD measurements, which are dominated by pial vein artifacts.^59,60^ By shifting the imaging contrast from venous oxygenation to microvascular volume, this approach enables measurement of neuronal activation at the mesoscopic scale, essential to advance our understanding of brain function and neurovascular coupling abnormalities that characterize many neurological conditions, including stroke and dementia.^73,74^

The layer-specific imaging capabilities of our bioengineered contrast agent open new avenues for understanding neurovascular disease mechanisms through quantitative assessment of the cortical layer function. FLH-RBCs indeed enabled voxel-wise estimation of the baseline blood volume fraction. Using a magnetic susceptibility correction of 0.15 ppm—aligned with the known susceptibility of Hbr at 0.157 ppm^50^—we achieved plausible values (*V*_f_ 2–3%, Hbt_0_ = 40–60 μM) with a clear gradient from surface to deep cortex. These estimates are more reliable than those derived from BOLD-based models, which lack direct sensitivity to microvascular volume and rather provide detailed insights into neurovascular architecture.

Future validation of FLH-RBC susceptibility through experimental calibration^44^ will strengthen its utility for quantitative CBV mapping and potentially improve sensitivity to early-stage neurovascular dysfunction.

### Implications for Translational Nanomedicine

Directly comparing free SPIONs (Ferucarbotran) and FLH-RBCs highlights the net clinical advantage of our method through the reduced dosage of iron required and modulation of pharmacokinetics. FLH-RBCs deliver effective contrast at 6.86 mg/kg iron, a concentration significantly below the 10–14 mg/kg doses commonly used for free SPIONs like Ferucarbotran or AMI-227.^34,75,76^ Despite lower iron load, FLH-RBCs achieve superior imaging performance through enhanced magnetic susceptibility optimization. Furthermore, our method maintains contrast for over 30 min, reaching a steady-state signal drop of ∼15% (at 10 μmoles Fe), unlike free SPIONs that are rapidly cleared by the liver and spleen.^26^ This improved circulation time supports the longitudinal monitoring of neurovascular disease progression and treatment efficacy, critical for many chronic conditions. The slower clearance reflects natural RBC turnover, which spans ∼120 days in humans, allowing for repeated imaging with minimal toxicity.^26^

The bioengineered contrast agent technique with optimized nanoparticle encapsulation process for human red blood cells used here leverages existing automated Red Cell Loader technologies developed by Erydel (now Quince Therapeutics), currently in Phase II/III trials for rare neurodegenerative disorders.^24,26,77^ These systems use hypotonic dialysis and resealing to load RBCs with therapeutic agents, enabling point-of-care blood processing with reinfusion of autologous, patient-matched carriers.

Our method requires only 50 mL of patient blood, processed over ∼2 h, enabling a personalized, immunologically safe diagnostic agent. Autologous delivery will also eliminate the size mismatch observed in our rat model using human RBCs (7–8 μm diameter, MCV ∼ 95 vs 6 μm, MCV ∼ 70 for rats), which may explain minor blood pressure changes postinjection.^23,30^ Importantly, our hypercapnic challenge data demonstrated that FLH-RBCs reached all brain areas without detectable vascular obstruction, indicating that the vasodilation induced by 10% increased FiCO_2_ facilitated their passage. While the use of non-native cells might be of concern in this case, it should be noted that all experiments were acute and performed under sustained anesthesia, with all animals surviving the full ∼6 h protocol (surgery and data collection) prior to being culled in accordance with the underpinning UK Home Office project license. Our results here detail the initial preclinical validation of efficacy for future testing in humans. Together, these findings establish FLH-RBCs as a biocompatible and generalizable nanocarrier platform that prolongs nanoparticle circulation, maintains magnetic performance, and enables high-resolution functional imaging. This biomimetic strategy is readily compatible with clinical MRI sequences and may be extended to other imaging modalities and therapeutic payloads, offering broad opportunities in neurology, oncology, and theragnostics.

Our bioengineered contrast agent’s versatility extends beyond conventional MRI to emerging imaging modalities crucial for comprehensive neurovascular assessment. Magnetic particle imaging studies (MPI) using Resovist- and Sinerem -loaded RBCs^78^ demonstrate the platform’s adaptability, with recent studies showing CBV measurements capabilities in rodents using functional MPI (fMPI) with free SPIONs.^79^ FLH-RBCs, with their extended half-life and encapsulation fidelity, could serve as a next-generation MPI tracer for fMPI, especially when combined with targeted therapeutic delivery, blurring the line between diagnosis and therapy. Integration with ultrahigh field MRI systems (≥7T) offers unprecedented resolution of neurovascular function across cortical layers, enabling detailed characterization of disease-specific laminar or vascular abnormalities. This positions FLH-RBCs as a powerful theragnostic tool capable of both monitoring and modulating disease process.

Some limitations remain. Anesthesia and physiological variability in animal models may affect hemodynamic responses. Future studies will incorporate multimodal imaging approaches, including concurrent high resolution intrinsic optical imaging spectroscopy (OIS)^34^ to measure hemodynamics in the form of total hemoglobin changes during stimuli, providing comprehensive validation of neurovascular responses and oxygen dissociation characteristics of labeled cells.

The use of human RBCs in rodents introduces size mismatches and should be addressed through autologous delivery in human trials. While encapsulation efficiency is reduced in mouse cells,^25,26^ the core principle remains transferable. Future studies will target neurovascular disease models in mice to assess diagnostic sensitivity in early Alzheimer’s or microvascular pathology.

Measuring FLH-RBC magnetic susceptibility directly and further optimizing loading concentrations will help improve contrast specificity and broaden diagnostic thresholds. It is also noted that blood is a colloidal suspension. Addition of SPIONs to the RBC pool may cause cell clumping. Future experiments should measure the zeta potential of FLH-RBCs as a crucial indicator of colloidal suspension stability. High zeta potential (either positive or negative) indicates that carriers repel each other, leading to a stable dispersion that resists settling or clumping. Across the literature, zeta values for native RBCs are reported between ∼−13.4 and −15.7 mV.^80,81^ This negative charge, due to the presence of sialylated glycoproteins in the cell member, prevents RBCs from aggregating. Sun et al. (2024) demonstrated that encapsulating cationic nanoparticles with a positive zeta 21.81 ± 3.65 mV can slightly decrease the RBC zeta potential and cause cell aggregation. Reducing the nanoparticle concentration to limit zeta potential drop to −11.9 ± 0.94 mV aggregation effects were avoided. We note that Ferucarbotran has a highly negative zeta potential, typically reported as −35 mV to −40 mV,^82^ due to its carboxydextran coating. It is therefore hypothesized that a stable RBC colloidal suspension is maintained. Although the zeta potential was not measured, the osmolarity of the mixtures was closely monitored throughout agent preparation. During the hypotonic dialysis, osmolarity is reduced (from 300 to 100 mOsm), which expands the electrical double layer and would increase the negative zeta potential as the cell swells. At the isotonic resealing stage, the osmolarity is restored to physiological levels (300 mOsm), and therefore it is reasonable to assume that the zeta potential of the FLH-RBCs returns to similar values of the native RBCs. Furthermore, the iron oxides are observed encapsulated within the FLH-RBCs in a homogeneous manner (see Figure 2) with no apparent adhesion to the cell membrane providing further indirect evidence that the zeta potential is similar to that of the native RBCs. Any increase in zeta potential would cause more repulsion between loaded cells, which would cause a reduction in blood hematocrit (HcT) level. In MRI, the transverse relaxation rate (*R*_2_*) is proportional to the HcT level.^50^ Reductions in HcT would lead to an increased MR signal. We note here that we observe a decrease in signal during neuronal activity, which is indicative that there is not a major change in the HcT level due to addition of the contrast agent. Improved blood gas analysis would confirm this.

## Conclusions

By leveraging the biocompatibility of autologous blood and the magnetic responsiveness of SPIONs, FLH-RBCs offer a scalable, safe, and high-fidelity alternative to conventional fMRI. This bioengineered blood-based contrast agent supports sensitive detection of CBV changes at laminar resolution, prolonged circulation for longitudinal studies, and integration with theragnostic platforms. FLH-RBCs have the potential to redefine functional brain imaging and transform the diagnostic landscape of neurovascular disease.

## Star Methods

### Methods

#### Ethics

Human blood was collected from healthy screened donors (*n* = 6; mean age 32; 4 female) at the Transfusion Centre of “S. Maria della Misericordia” Hospital in Urbino (PU), Italy, following appropriate institutional review board approval. The study obtained informed consent from participants and followed the principles of ethical human research outlined in the Declaration of Helsinki.

Following preparation, Ferucarbotran-loaded human red blood cells (FLH-RBCs) samples were shipped to the UK for preclinical testing.

All preclinical procedures involving animals were carried out in compliance with the UK Animals (Scientific Procedures) Act 1986 and were authorized by the UK Home Office. All experimental protocols were reviewed and approved by the University of York Animal Welfare and Ethical Review Body (AWERB) and were conducted in licensed facilities by appropriately trained and licensed personnel. Female hooded Lister rats (*n* = 7; 3–6 months old) weighing 200–250 g were kept in a 12 h dark/light cycle, at a constant temperature of 22 °C, with food and water ad libitum.

#### Encapsulation of SPIONs in Human RBCs

Human blood (15 mL per donor, *n* = 6) was collected in heparinized vacutainers. RBCs were isolated by centrifugation and washed in a Hepes buffer. For SPION encapsulation, cells were subjected to hypotonic dialysis in the presence of Ferucarbotran (11.2 mg­(Fe) mL^–1^) at 4 °C for 75 min, followed by isotonic resealing at 37 °C for 45 min, thus obtaining SPION-loaded human RBCs (FLH-RBCs). Final suspensions were adjusted to 44% hematocrit (HcT). Unloaded (UL-RBCs; *n* = 3) controls were prepared following the same dialysis procedure but in the absence of the SPIONs. In all cases (*n* = 6), ∼3 mL of the blood donation was set apart and the samples were used as nondialyzed (ND-RBCs) samples for baseline cell integrity measures. A schematic of the procedure is shown in Figure 1. Full loading protocols are provided in the Supporting Information Methods.

#### Cell Integrity

To determine whether the FLH-RBCs retained the biological properties of native cells, several features of cell integrity were examined.^78^ Mean corpuscular volume (MCV), mean hemoglobin concentration (MCH), and mean corpuscular hemoglobin concentration (MCHC) were measured with an automated hemocytometer (Model MICROS O.T, Horiba ABX Diagnostics, Italy). We evaluated the number of total intact erythrocytes before and after Ferucarbotran loading to determine the percentage of cell recovery.

#### NMR Measurements

The *T*_1_ longitudinal relaxation times of FLH-RBCs samples were measured at 9.4T (Avance-400 NMR, Bruker) and used to estimate the iron concentration.^29^ The values of (1/*T*_1_^*c*^ – 1/*T*_1_^0^) (where *T*_1_^*c*^ is the relaxation time at the concentration [*c*] of contrast agent and *T*_1_^0^ the relaxation time of the RBCs sample without the contrast agent) were plotted versus the concentration of Fe in Ferucarbotran (0 mM< [*c*] < 18 mM) and were fitted by a least-squares method to a straight line, the slope of which is the longitudinal relaxivity (*r*1 = 1.3003 s^–1^ mM^–1^). *T*_2_ was measured using the Carr–Purcell–Meiboom–Gill method (CPMG). The transverse relaxivity (*r*2) was calculated in a similar way by plotting the values of (1/*T*_2_^*c*^ – 1/*T*_2_^0^) versus [*c*] resulting in *r*2 = 87.228 s^–1^ mM^–1^.^27^ These values are then used to estimate the concentration of Ferucarbotran encapsulated.

#### Optical Microscope Observations

Light microscopy images (Figure 2) were obtained using a Nikon Eclipse 80i microscope (Nikon Instruments, EuropeBV, Kingston, Surrey, England). 10 ml of RBCs were fixed with glutaraldehyde and a small drop was placed on a glass slide and then covered with a coverslip. All samples were observed using a 20x objective to check the quality of the RBCs before proceeding with a Transmission electron microscopy (TEM) embedding procedure.

#### Transmission Electron Microscopy (TEM)

TEM analysis was conducted on ultrathin (60 nm sections obtained using an MT-X ultratome; RMC; Tucson, AZ, USA) of glutaraldehyde- and OsO_4_-fixed RBC samples embedded in epoxy resin. Ultrastructural characterization was performed using either a CM10 or a CM200 Philips transmission electron microscope (FEI-Philips, Hillsboro, OR, USA) equipped with a LaB6 source, compustage goniometer and double-tilt sample holder. Analysis of whole-RBCs was also performed on glutaraldehyde-fixed cells dropped onto a Formvar-coated copper grid and contrasted with cold carboxymethyl-dextran. The whole-RBCs grids were analyzed by a JEOL JEM-1011 TEM operating at 100 keV.

#### Elemental Analysis

The iron concentrations were measured by elemental analysis using an inductively coupled plasma atomic emission spectrometer (ICP-OES Varian 720-ES). Before analysis, the RBC samples were digested in a concentrated HCl/HNO_3_/H_2_O_2_ 3:1:1 (*v*/*v*) solution for 24 h. For evaluating the presence of superparamagnetic nanoparticles, the iron concentration determined in the UL-RBC sample has been subtracted from the FLH-RBCs.

#### Preclinical fMRI

The preclinical fMRI setup used a nonrecovery model and is shown in Figure 1. All animals (*n* = 7) were anesthetized (i.p., urethane 1.25 g/kg) with additional 0.1 mL doses administered as necessary (depth assessed via pinch reflex). Complementary analgesia was administered (sc Buprenorphine, 0.02–0.05 mg/kg). Animals were tracheotomized to allow artificial ventilation (at 70 breaths per minute, Vt 3 mL V/min = 210, PEEP 3 cm H_2_O, VentElite, Harvard Apparatus, US) with medical grade air (BOC, UK). Rectal temperature was maintained and monitored at 37 °C throughout surgical procedures using a heat pad (TC-1000 Temperature Controller, CWE Inc., US). Femoral arteries and veins were cannulated for contrast infusion and blood pressure monitoring (maintained between physiological limits, MABP, 100–110 mmHg,^83^ via infusion of Phenylephrine; 0.13–0.26 mg/h). Electrical stimulation was delivered via tungsten electrodes, insulated to within 2 mm of the tip and inserted between whisker rows A/B and C/D of the left whisker pad.

Preclinical MRI measurements were made at 7 T (Bruker BioSpec 70/30, AVANCE III, 310 mm bore Bruker Biospin GmbH, Ettlingen, Germany) with preinstalled BGA20S gradient system (300 mT/m, Bruker Biospin GmbH, Ettlingen, Germany). A 1H quadrature coil was used for RF transmission (300 1H 112/086 QSN TO AD, Model no: 1P T12053 V3, Bruker Biospin GmbH, Ettlingen, Germany) and a 4-channel rat brain array coil for reception (RF ARR 300 1H R.BR. Two × 2 RO AD, Model no: 1P T11483 V3, Bruker Biospin GmbH, Ettlingen, Germany). Structural images (Gradient Echo FLASH) guided positioning of axial GE-Echo Planar Imaging (EPI) scans (TR/TE = 1000/12 ms, 96 × 96, FOV = 30 × 30 mm^2^, slice thickness = 1 mm, FA = 90°, slices = 5, dummy scans = 10; Figure 1).

BOLD signal changes were obtained (across all *n* = 7 subjects) in response to whisker stimulation (60 s baseline followed by 16 s, 7 trials, 96 s ISI at 1.2 mA, 5 Hz) and/or respiratory challenge, consisting of a 1 min baseline (medical air, 4l/min), 10% increased FiCO_2_ for 2 min, and a further 2 min rest period (medical air—4l/min).

Three rats received 1.5 mL of FLH-RBCs (11.27 mM ± 2.79 of Fe) via femoral vein for the purpose of calculating blood volume fraction (see Calculation of Blood Volume Fraction below). Functional experiments were repeated after the full injection of the contrast agent. To account for BOLD contributions, the functional signals pre- and postcontrast injection were normalized (see Quantification of CBV below).

Control animals received either 1.5 mL of UL-RBCs (*n* = 3) and repetition of functional measures or 0.4 mL of free SPIONs (*n* = 1). In the free SPION control group, 10 min of GE-EPI was used to observe contrast agent washout followed by 256 × 256 GE structural scans captured for up to 80 min post injection. Data were used to estimate blood circulation half-life in comparison to the FLH-RBCs.

#### fMRI Data Analysis

All MRI data analysis was carried out in MATLAB (The MathWorks inc.) using an in-house code (available upon request). GE-EPI images were registered with structural scans. A general linear model with DC, ramp, and boxcar stimulus regressors was used to generate activation maps. A region of interest (ROI) was defined by thresholding *z*-score maps and extracting the time series. Detrending over the experimental run removed the ramp components, and a suitable Savtzky-Golay filter was applied to the mean time series across pixels to filter out high frequency noise. For each animal, and each experimental condition, a mean time series pre- and postcontrast injection was calculated.

#### Quantification of CBV

Changes in MR signal due to susceptibility arising from contrast agents were modeled according to established equations for contrast-enhanced CBV imaging.^44,47,84^ Equations for absolute and fractional CBV changes (including Manderville et al. (1998)^85^ model) were applied using signal intensities pre- and postcontrast. Calculation formulas and susceptibility assumptions are provided in the Supporting Information.

#### Cortical Profiles

To assess the spatial specificity of the BOLD and CBV responses, a 2D cortical depth profile is created from the *z*-score map of the associated analyzed GE-EPI data. A line perpendicular to the cortical surface (defined from high resolution structure scans) is extended 3 mm into the brain. *Z* scores across a 4 mm wide box are averaged to form the profile.

#### Calculation of Blood Volume Fraction

Baseline blood volume fraction was determined using stepwise FLH-RBCs infusion and voxel-wise mapping of ΔR_2_*. Blood volume fraction (*V*_f_) maps were generated from the ΔR_2_* maps following Tropres et al. (2001)^44^ and can be converted to baseline blood volume (Hbt_0_) using physiological parameters such as the rat hematocrit fraction (Hct), the cerebral tissue to large vessels ratio (*R*_*c*/l_), the concentration of hemoglobin in red blood cells ([Hb]_RBC_), and the molecular mass of hemoglobin (Mm_Hb_). Quantification formulas are detailed in the Supporting Information Methods.

## Supplementary Material


