# Supplementary material for: Red Blood Cell-Encapsulated Nanoparticles for Long-Circulating, Improved Specificity Functional MRI
Source: Chem Biomed Imaging. 2026 Jan 12;4(6):1139–52. doi: 10.1021/cbmi.5c00190 (PMC13291955; doi:10.1021/cbmi.5c00190)
Supplement: Supplementary file 1 [file im5c00190_si_001.pdf]

Supporting Information

Red Blood Cell–Encapsulated Nanoparticles for Long-Circulating, Improved Specificity Functional MRI.

*Elizabeth J Fear[1,2,3] ‡, Antonella Antonelli[1] ‡, Pasant Abdalla[1], Isaac Watson[4], Riccardo Di Corato[5,6], Victoria Annis[7], Oliver J Mundell[8,9], Simon B Duckett[7], Luigia Rossi[1], Elisa Zamboni[10], Mauro Magnani[1] and Aneurin J Kennerley[8,9]**

AUTHOR ADDRESSES

[1]Department of Biomolecular Sciences, University of Urbino Carlo Bo, 61029 Urbino, Italy

[2]Department of Neurosciences, Imaging and Clinical Sciences, University "G. D'Annunzio" of Chieti-Pescara, Chieti, Italy

[3]Institute for Advanced Biomedical Technologies, University "G. D'Annunzio" of Chieti-Pescara, Chieti, Italy

[4]Biomedical Imaging Science Department, Leeds Institute of Cardiovascular and Metabolic Medicine, University of Leeds, Leeds, LS2 9JT, UK

[5]Institute for Microelectronics and Microsystems (IMM), CNR, Via Monteroni, Lecce 73100, Italy

[6]Center for Biomolecular Nanotechnologies, Istituto Italiano di Tecnologia, Arnesano 73010, Italy

[7]Department of Chemistry, University of York, York, YO10 5DD, UK

[8]Department of Sports & Exercise Science, Manchester Metropolitan University, Manchester, M15 6BH, UK

[9]Wolfson ACTIVE Imaging Laboratory, Institute of Sport, Manchester Metropolitan University, Manchester, M15 6BH, UK

[10]School of Psychology, University of Nottingham, Nottingham, NG7 2RD, UK

*Corresponding author email: A.Kennerley@mmu.ac.uk

SUPPORTING INFORMATION RESULTS

Representative Multi-Slice fMRI Data –Whisker Stimulation

Supporting Information Figure S1 shows representative data of the BOLD signal found in the whisker barrel cortex and distribution across the 5 slices in response to somatosensory whisker stimulation (16 s, 1.2mA, 5 Hz). The BOLD signal was identified in four of the five slices measured and ranges from 1 to 2.5 %.

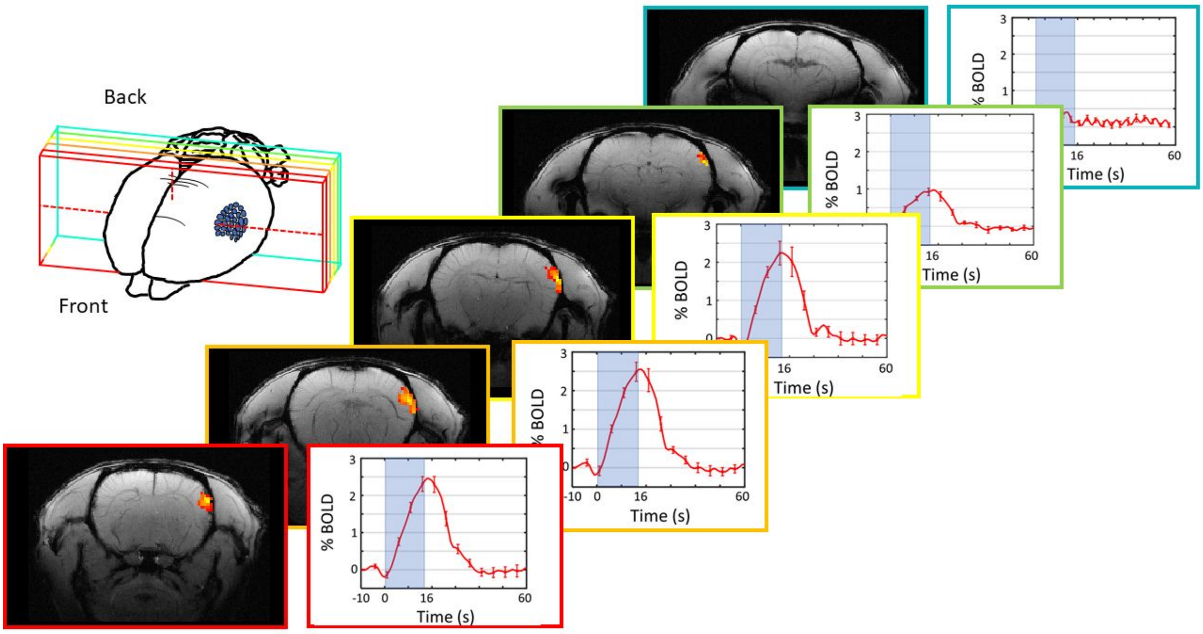

Supporting Information Figure S1. fMRI responses to somatosensory whisker stimulation.
fMRI measures in response to somatosensory whisker stimulation (16s, 1.2mA, 5Hz) showing representative BOLD signal maps overlaid on a GE structural scans and the mean positive BOLD time series for each of the 5 slices measured.

Supporting Information Figure S2 shows representative data of the negative fMRI signal in the whisker barrel cortex and distribution across the 5 slices found after injection of FLH-RBCs. The negative signal was identified in all of the five slices measured and ranges from -1 to -3.8 %. FMRI data using whisker stimulation showed an average BOLD signal prior to injection of FLH-RBCs of (2.5%) and an average signal after injection of FLH-RBCs (2.5% - 3.5%). These values correspond to a signal increase of ~9% (21 - 25%) following normalization by baseline as per **Equation 5** (main paper).

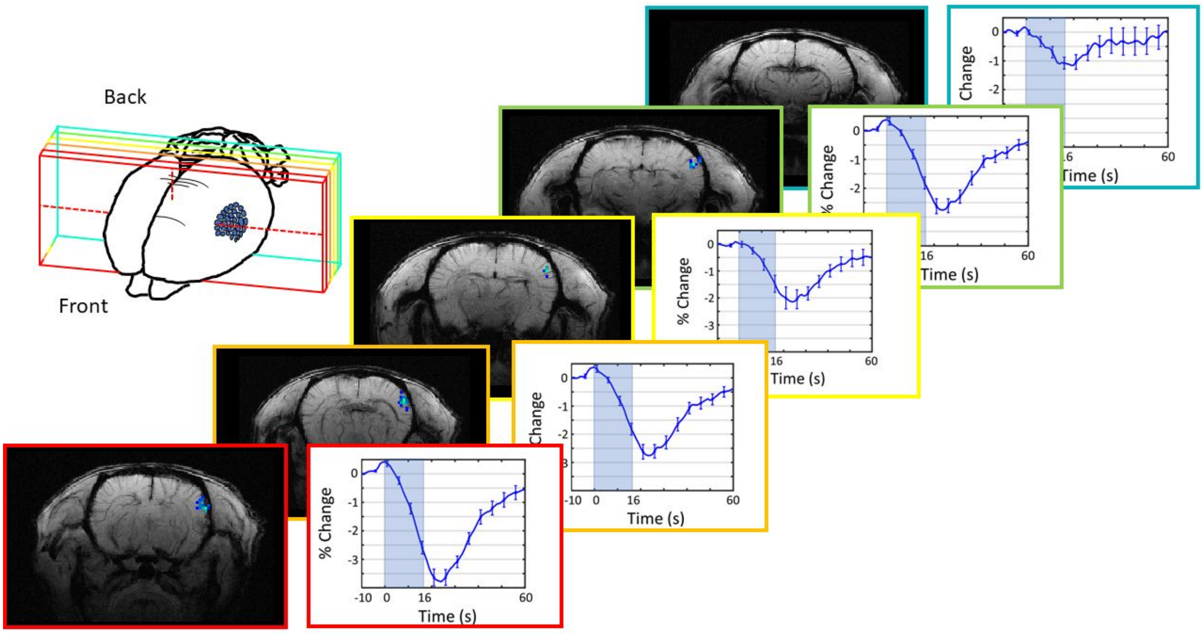

Supporting Information Figure S2. fMRI responses to somatosensory whisker stimulation post FLH-RBCs injection.
fMRI measures in response to somatosensory whisker stimulation (16s, 1.2mA, 5Hz) showing representative signal maps after injection of FLH-RBCs (rat2 16.4 mM of FLH-RBCs) overlaid on a GE structural scans and the resulting negative time series for each of the 5 slices measured.

Figure 4 (main paper) shows the calculated corresponding CBV volume change in the whisker barrel area using the pre (BOLD) and post (Fe) experiments. CBV volume change was identified in all five slices measured and ranges from 6-12% (compared to the baseline CBV). These values are consistent with previously reported ΔCBV estimates.

Rat 2 – Hypercapnia respiratory challenge.

Supporting Information Figure S3 shows representative fMRI data obtained after hypercapnia respiratory challenge (increased end-tidal $FiCO_2$ <10%) and a BOLD response ranging from 5-7% (8-10%) found across the whole brain and distributed in all 5 slices measured.

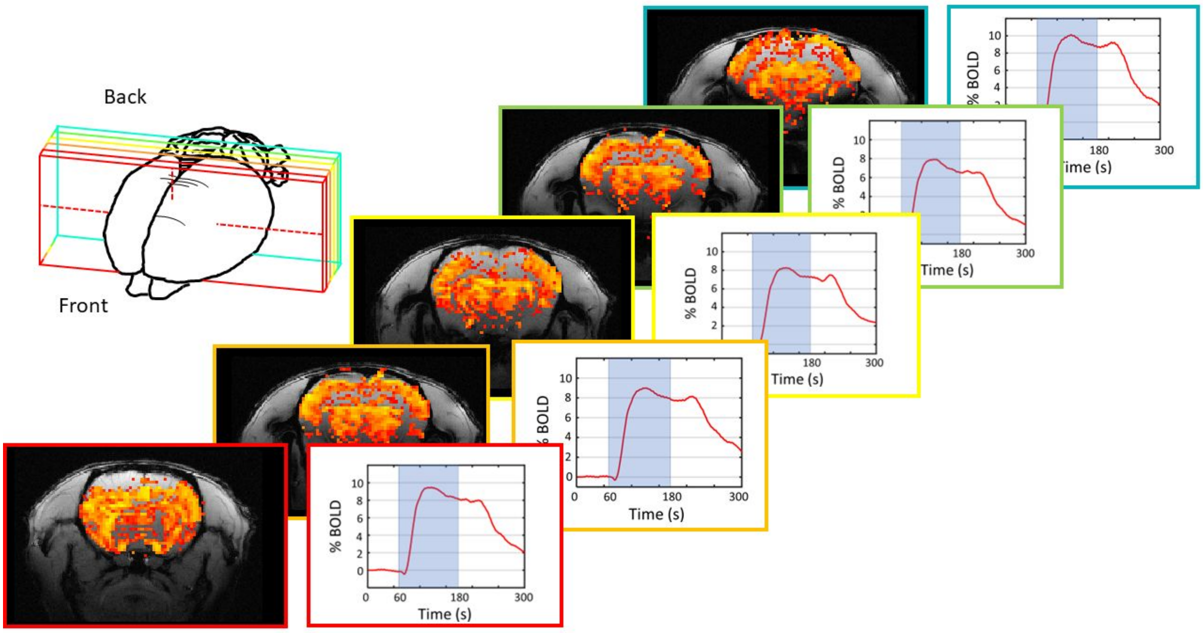

Supporting Information Figure S3. fMRI responses to hypercapnia challenge.
fMRI measures in response to hypercapnia respiratory challenge (increased end-tidal FiCO$_2$ *<10%) showing representative BOLD signal maps overlaid on GE structural scans and the positive BOLD time series for each of the 5 slices measured.*

Supporting Information Figure S4 shows representative data highlighting the negative fMRI signal across the whole brain in response to hypercapnia respiratory challenge and its distribution across the 5 slices found after injection of FLH-RBCs. Responses ranged from -10 to -6%. Using an average BOLD response of ~9%, and an average signal after injection FLH-RBCs of -8% this would correspond to a signal increase of ~70% (following **Equation 5**, main paper).

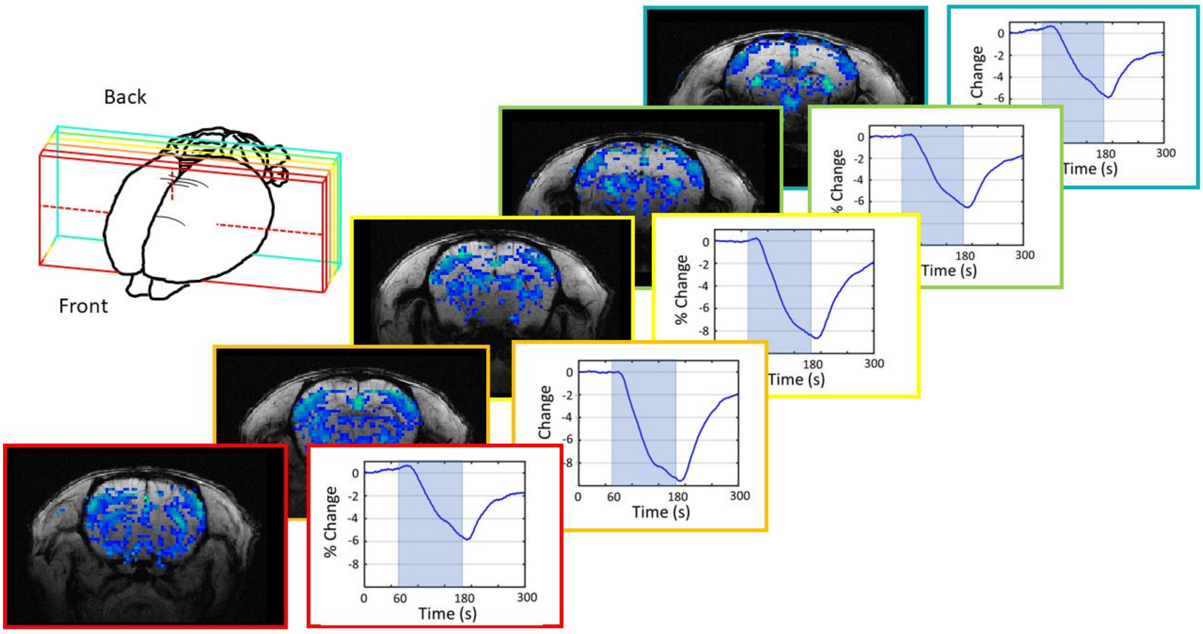

Supporting Information Figure S4. fMRI responses to hypercapnia challenge post FLH-RBCs injection.
fMRI measures in response to hypercapnia respiratory challenge (increased end-tidal FiCO$_2$ *<10%) showing representative signal maps after injection of FLH-RBCs (rat2 16.4 mM of FLH-RBCs) overlaid on GE structural scans and the resulting negative time series for each of the 5 slices measured.*

The corresponding CBV volume change across the whole brain after hypercapnia respiratory challenge was calculated using the pre (BOLD) and post (Fe) experiments. CBV volume change was identified in all five slices measured and was in the range of 40-60% (**Supporting Information Figure S5**).

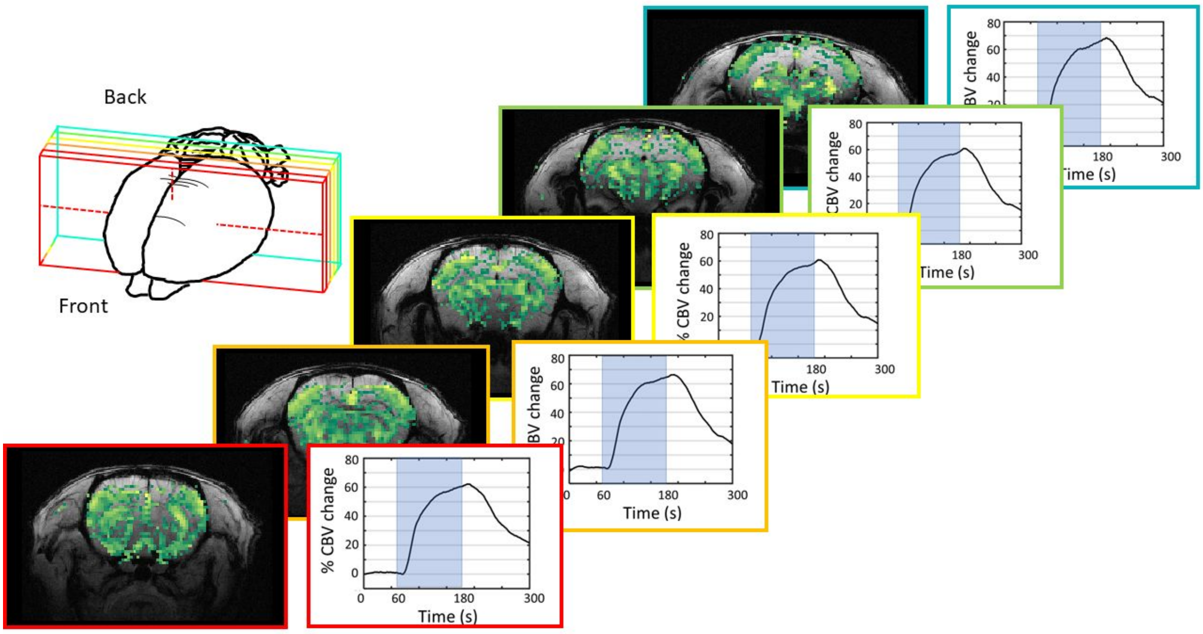

Supporting Information Figure S5. Changes in CBV measures following hypercapnia challenge. *fMRI measures in response to hypercapnia respiratory challenge (increased end-tidal $FiCO_2$ <10%) showing %CBV changes overlaid on GE structural scans and the positive %CBV time series for each of the 5 slices measured after injection of FLH-RBCs (rat2 16.4 mM of FLH-RBCs).*

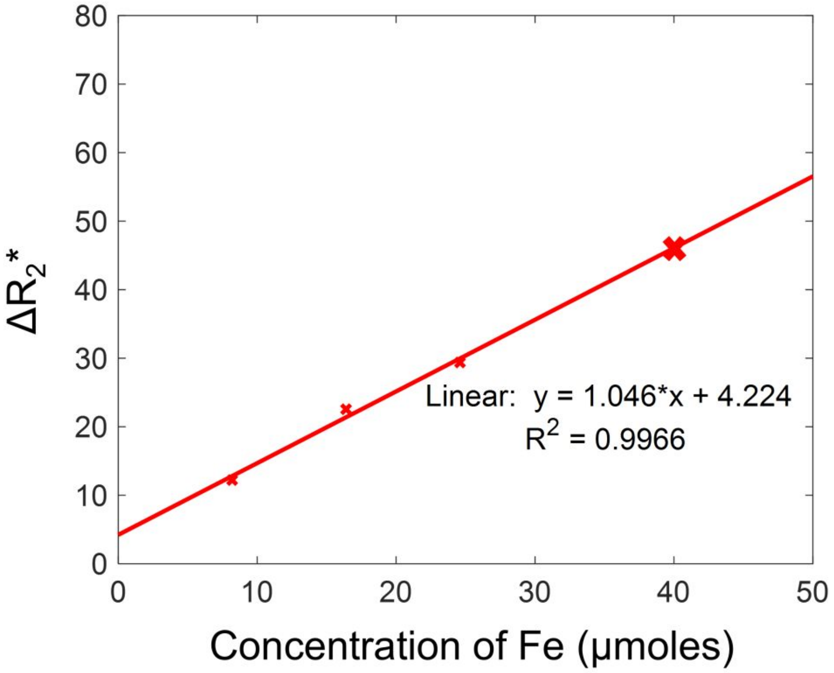

Supporting Information Figure S6. Relationship between ΔR2* and FLH-RBCs.
ΔR_2^* versus concentration of infused FLH-RBCs experimental data points (0 to 1.5 ml, total 24.6 µmoles, red) extrapolated to 40 µmoles of Fe/200 g rat in line with Tropres et al. (red bold cross).

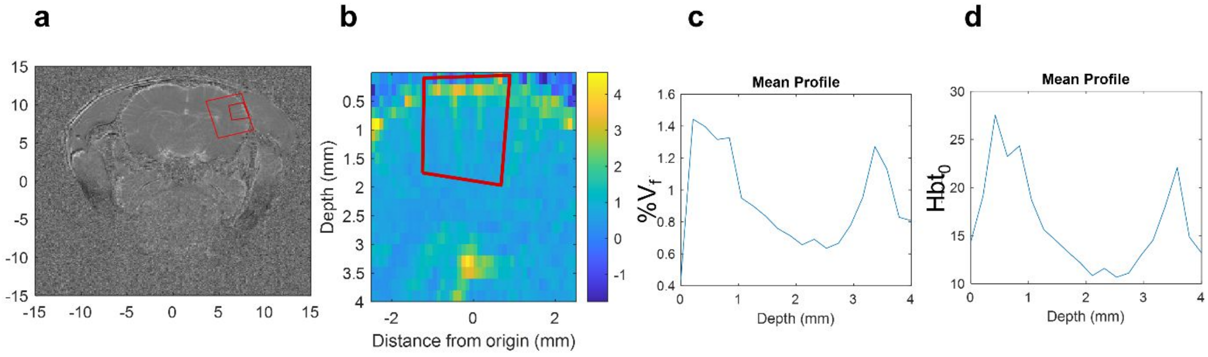

Supporting Information Figure S7. Blood volume fraction and concentration maps with FLH-RBCs.
Blood volume fraction (V_f) and concentration (Hbt_0) maps with FLH-RBCs (16.4 mM) using magnetic susceptibility value $\Delta\chi$ = 0.571 ppm (a-c). (a) Mean V_f map per voxel. (b) V_f calculated across a 2D cross section of whisker barrel cortex region. (c) Mean profile of the 2D cross section showing the mean V_f. (d) Mean profile of the 2D cross section showing the mean Hbt_0 per voxel across the cortex.

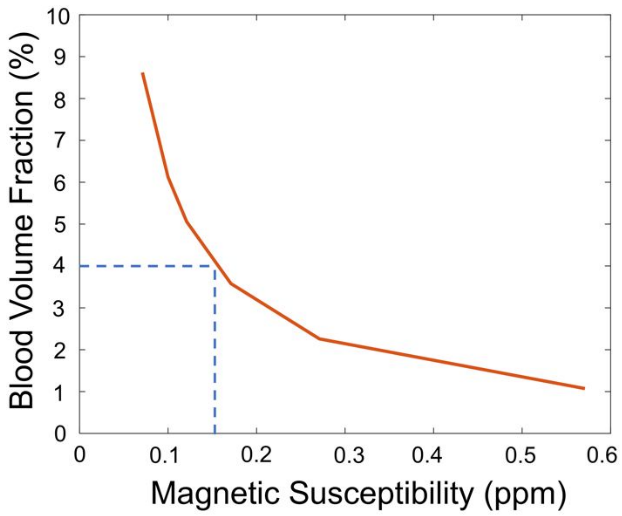

Supporting Information Figure S8. Blood volume fraction as a function of magnetic susceptibility for FLH-RBCs.
Calculated blood volume fraction in the cortex as a function of magnetic susceptibility with infusion of 1.5 ml of FLH-RBCs, 16.4 mM, total 24.6 µmoles of Fe.

STAR★METHODS

KEY RESOURCES TABLE

REAGENT OR RESOURCE	SOURCE	IDENTIFIER
Biological samples		

Human blood	Healthy adults, n=6; Transfusion Centre of “S. Maria della Misericordia” Hospital in Urbino (PU), Italy.	N/A
Ferucarbotran® iron oxides nanoparticles	Meito Sangyo Co., Ltd. Nagoya Research Lab., Japan	Resovist, SH U 555 A
Deposited data		
Raw and analysed data	This paper	Mendeley: http://dx.doi.org/10.xxxxx
Experimental models: Organisms/strains		
Rat: Hood Lister<br>Female; 160-220 grams.<br>Age ~70-76 days old	Charles River, UK.	911 – Hooded Lister Rats
Software and algorithms		
MATLAB	MathWorks	https://www.mathworks.com/products/matlab.html
Spike2	CED, Cambridge Electronic Design Limited	https://ced.co.uk/products/spkovin
Other		
Haemocytometer	Horiba ABX Diagnostics, Italy	MICROS O.T.
9.4 Tesla NMR scanner	Bruker Biospin GmbH, Ettlingen, Germany	Avance-400 NMR
Microscope	Nikon Instruments, EuropeBV, Kingston, Surrey, England	Nikon Eclipse 80i
MT-X ultratome	RMC; Tucson, AZ, USA	MT-X ultratome
Transmission Electron Microscope	FEI-Philips, Hillsboro, OR, USA	CM10 / CM200 Philips
Transmission Electron Microscope		JEOL JEM-1011, operating at 100 KeV
Inductively coupled plasma atomic emission spectrometer		ICP-OES Varian 720-ES
Heat pad	CWE Inc, US	TC-1000 Temperature Controller
Waterbed MRI inset	Thermo Scientific, US	Haake SC100/S 5P
Artificial ventilation	Harvard Apparatus, US	VentElite
Syringe pump	Kent Scientific Corporation, US	
Pressure transducer	World Precision Instruments, US	Pressure Monitor BP-1
Micro1401	CED, Cambridge, UK	Micro1401
7 Tesla scanner	Bruker Biospin GmbH, Ettlingen, Germany	Bruker BioSpec 70/30, AVANCE III, 310 mm bore
Gradient system	Bruker Biospin GmbH, Ettlingen, Germany	BGA20S gradient system (300 mT/m)
1H quadrature coil for RF transmission	Bruker Biospin GmbH, Ettlingen, Germany	300 1H 112/086 QSN TO AD, Model no: 1P T12053V3
4-channel rat brain array coil for reception	Bruker Biospin GmbH, Ettlingen, Germany	RF ARR 300 1H R.BR. 2x2 RO AD, Model no: 1P T11483V3

SUPPORTING INFORMATION METHODS

Encapsulation of SPIONs in Human RBCs

Human blood (15 mL per donor, n = 6) was collected in heparinized vacutainers. RBCs were isolated by centrifugation at 1400g at 4 °C for 10 min from freshly drawn blood. The serum and buffy coat were removed, and the packed cells were washed three times with Hepes buffer (10 mM Hepes, 140 mM NaCl, 5 mM glucose, pH 7.4) and then resuspended in the same buffer at 70% hematocrit (HcT). For loading (n = 3), cells were dialyzed in the presence of magnetic nanoparticles (at 11.2 mg(Fe).ml^{-1}) for 75 min using a tube with a 12-14 kDa cut-off in 50 vol of a hypotonic buffer (10 mM $NaHCO_3$, 10 mM NaH_2PO_4, 20 mM glucose, and 4 mM $MgCl_2$ at pH 7.4), containing 2 mM ATP and 3 mM reduced glutathione.[38,39,42] Iron oxides nanoparticles with a mean hydrodynamic diameter of 57 nm; [56 mg Fe/ml or 1M Fe] from Meito Sangyo Co., Ltd. Nagoya Research Lab., Japan were used. The osmolarity of the dialysis buffer was 70 ± 2 mOsm. All these procedures were performed at 4 °C under sterile conditions. Resealing of RBCs was achieved by adding 10% by volume of PIGPA (5 mM adenine, 100 mM inosine, 2 mM ATP, 100 mM glucose, 100 mM sodium pyruvate, 4 mM $MgCl_2$, 194 mM NaCl, 1.606 M KCl, and 35 mM NaH_2PO_4 at pH 7.4) per vol of dialyzed RBCs and by incubating at 37°C for 45 min. The resealed cells were recovered by centrifugation at 400g and washed four times with Hepes buffer to remove the non-encapsulated magnetic nanomaterial and resuspended at 44% HcT to be ready for in-vivo infusion.

It is noted that unloaded (UL)-RBCs (n = 3) were prepared following the same dialysis procedure but in the absence of the SPIONs.

In all cases ~3 ml of the blood donation was set apart and RBCs washed and re-suspended at 44% Hct in Hepes buffer. These samples were used as non-dialysed (ND)-RBCs for 'before' cell integrity measures.

NMR measurements

The T_1 longitudinal relaxation times of FLH-RBCs samples were measured at 9.4T (Avance-400 NMR, Bruker) and used to estimate the iron concentration[49]. A 180°-τ-90° inversion recovery pulse sequence with 10 inversion times (τ) and a fixed relaxation delay of at least 5 × T_1 was used.

The values of ($1/T_1^c$-$1/T_1^0$) (where T_1^c is the relaxation time at the concentration [c] of contrast agent and T_1^0 the relaxation time of the RBCs sample without the contrast agent) were plotted versus the concentration of Fe in Ferucarbotran® (0 mM< [c] < 18 mM) and were fitted by a least squares method to a straight line, the slope of which is the longitudinal relaxivity ($r1$ =1.3003 sec^{-1} mM^{-1}). T_2 was measured using the Carr-Purcell-Meiboom-Gill method (CPMG). Ten echo-times were chosen based on an estimated T_2 value. The transverse relaxivity ($r2$) was calculated in a similar way by plotting the values of ($1/T_2^c$-$1/T_2^0$) versus [c] resulting in $r2$=87.228 sec^{-1} mM^{-1}.[42] Consequently, it was possible to estimate the concentration of Ferucarbotran® encapsulated in the erythrocytes by using the inverse formula [c]=$1/T_1^c$ -$1/T_1^0$ /$r1$ and [c]=$1/T_2^c$-$1/T_2^0$ /$r2$ in correspondence of the measured T_1^c and T_2^c values of loaded RBC suspensions at 44% hematocrit.

Quantification of CBV

Changes in MR signal due to susceptibility arising from contrast agents can be described by **Equation 1**.[13,22]

$$\frac{S(c')}{S(c=0)} = \frac{S_{rest}e^{-\frac{4}{3}\pi\gamma B_0 \Delta\chi(c')CBV\ TE}}{S_{rest}e^{-\frac{4}{3}\pi\gamma B_0 \Delta\chi(c=0)CBV\ TE}} \qquad [1]$$

Where S is the MR signal, γ is the gyromagnetic ratio (2.68 e^{8}rad.s$_{-1}$T$_{-1}$), B_0 is the static magnetic field (7T) and $\Delta\chi$ is the magnetic susceptibility difference at SPION concentration c. S_{rest} (ρ,T2*,etc.) contains signal parameters that are independent of the iron oxide contrast agent injected, c is the relative dose of contrast agent in randomly orientated cylindrical vessels[51] and it is assumed that CBV≪1, and T_E≫1δω.[22] CBV refers to the relative blood volume in units of ml per ml of tissue. Contrast agent induced susceptibility change based on iron content Δχ(c′) can be taken from the literature.[22,47]

By considering the change in relaxation rate induced by the agent, c':

$$\Delta R_2^*(c') \approx \frac{4}{3}\pi\gamma B\Delta\chi(c')CBV \qquad [2]$$

and assuming that $\Delta R_2^* \ll TE$ the measured MR signal can be converted into absolute CBV using **Equation 3**

$$CBV = \frac{ln\left(\frac{S_{pre}}{S_{post}}\right)}{-\frac{4}{3}\pi\gamma B\Delta\chi(c)\ TE}$$

[3]

With absolute changes in CBV between active and rest conditions estimated by:

$$\Delta CBV = CBV_{act} - CBV_{rest} = \frac{1}{\frac{4}{3}\pi\gamma B_0\Delta\chi(c)}\left[ln\left(\frac{S_{pre}^{act}}{S_{post}^{act}}\right) - ln\left(\frac{S_{pre}^{rest}}{S_{post}^{rest}}\right)\right] \qquad [4]$$

And following Manderville et.al. (1998)[52] fractional changes in CBV estimated using:

$$\frac{CBV}{CBV_0} = \frac{\ln[\frac{S_{post}^{act}}{S_{pre}^{rest}}]}{\ln[\frac{S_{pre}^{act}}{S_{pre}^{rest}}]} \qquad [5]$$

Where S^{rest} is the baseline signal (e.g. first 60s of each run), S^{act} is the signal during neuronal activation or respiratory challenge, and pre/post denotes the signal before and after FLH-RBCs /contrast agent injection. The relative CBV signal was then calculated as a fractional change from the 1-minute baseline.

Calculation of Blood Volume Fraction

At various stages during the infusion (e.g. every 0.5 ml of FLH-RBCs up to 1.5 ml) high resolution Gradient Echo (GE) T_2* weighted images were acquired in the axial plane for the assessment of baseline blood volume fraction[22] (256 × 256 pixels, FOV=30 × 30 mm, slice thickness = 1 mm, slices = 9, TR/TE = 1000/12 ms, flip angle = 90°, 2 averages).

On a pixel-by-pixel basis, ΔR_2^* maps at each concentration of FLH-RBCs were created using **Equation 6**.

$$\Delta R_2^*(c) = -ln\left(\frac{S_0(c)}{S_0(0)}\right)/TE \qquad [6]$$

Where $S_0(0)$ is the baseline signal without the FLH-RBCs, $S_0(c)$ is the baseline signal at dose 'c' and TE is echo time. Blood volume fraction (V_f) maps were generated from the ΔR_2^* maps following Tropres et al.(2001).[22] GE structural scans were used to calculate the blood volume fraction maps, where ΔR_2^* is found as a function of FLH-RBCs concentration, c, per pixel. Blood volume fraction V_f was calculated using **Equation 7**.

$$V_f = \frac{3\ \Delta R_2^*(c)}{4\pi\gamma B_0\Delta\chi(c)} \qquad [7]$$

Where γ is the gyromagnetic ratio (2.68 e^8rad.s^{-1}.T^{-1}), B_0 is the static magnetic field (7T) and $\Delta\chi$ is the susceptibility difference between blood with and without FLH-RBCs at concentration c. The gradient

of the plot of ΔR_2^* against the FLH-RBCs dose can be extrapolated back to 200 μM/kg, where a susceptibility of $\Delta\chi$ = 0.571ppm (Topres et al. [22]) used in **Equation 7**, gives a measure of the baseline blood volume fraction. The blood volume fractions can be converted into baseline blood volume (Hbt_0) by:

$$Hbt_0 = V\ Hct\ R_{c/l} \frac{[Hb]_{RBC}}{Mm_{Hb}} \quad [8]$$

Where Hct is the rat haematocrit fraction (0.46), $R_{c/l}$ is the ratio of cerebral tissue to large vessels (0.69), $[Hb]_{RBC}$ is the concentration of haemoglobin in red blood cells (340g) and Mm_{Hb} is the molecular mass of haemoglobin (64450g/M).
